# Supplementary material for: Concurrent measurement of working memory and inhibitory control and their correlations with autistic and ADHD traits in the general population
Source: PLoS One. 2026 Jan 5;21(1):e0339846. doi: 10.1371/journal.pone.0339846 (PMC12768290; doi:10.1371/journal.pone.0339846)
Supplement: S1 Appendix — (DOCX) [file pone.0339846.s001.docx]

**S1 Appendix: Accuracy in the flanker task and spatial conflict task in Study 1**

**S1a) Accuracy in the flanker task**

Descriptive statistics for participants’ accuracy in the flanker task are shown in Table S1.1.

**Table S1.1. Descriptive statistics of accuracy (proportion correct) in the flanker task (N=96).**

| Condition | Mean | Std. Deviation | Minimum | Maximum |
| --- | --- | --- | --- | --- |
| Low memory and congruent | .961 | .040 | .822 | 1 |
| Low memory and incongruent | .931 | .061 | .628 | 1 |
| High memory and incongruent | .941 | .077 | .317 | 1 |
| High memory and congruent | .935 | .076 | .378 | 1 |

A Bayesian repeated-measures ANOVA on flanker task accuracy revealed that the best-fitting model included the main effects of memory load and congruency, as well as their interaction (BF₁₀ = 5468.498 against the null model). The analysis provided strong evidence for including a congruency effect (BF₍incl₎ = 198.392), with higher accuracy on congruent trials (M = .952, SD = .045) than on incongruent trials (M = .933, SD = .055). There was also strong evidence for including the memory load × congruency interaction (BF₍incl₎ = 74.631). However, there was evidence against including a main effect of memory load (BF₍excl₎ = 2.697).

To decompose the interaction, separate Bayesian ANOVAs were conducted. For congruent trials, there was evidence for a memory load effect, as the model including memory load was preferred over the null model (BF₁₀ = 4.147). In contrast, for incongruent trials, there was evidence against a memory load effect, with the null model preferred (BF₀₁ = 5.608). Under low memory load, there was strong evidence for a congruency effect (BF₁₀ = 35,930), whereas under high memory load, the data instead supported the absence of a congruency effect (BF₀₁ = 4.266). Accuracy across all conditions is displayed in Fig S1.1.

**Fig S1.1. Accuracy (proportion correct) in the flanker task.** Error bars indicate ±1 standard error of the mean (SEM).





**S1b) Accuracy in the spatial conflict task**

Table S1.2 presents descriptive statistics for participants' accuracy in the spatial conflict task. A Bayesian repeated-measures ANOVA on accuracy in the spatial conflict task showed that the best-fitting model included the main effects of memory load and congruency (BF₁₀ = 415,648.672 relative to the null model). There was weak evidence for including a memory effect (BF₍incl₎ = 1.246) and strong evidence for including a congruency effect (BF₍incl₎ = 343,518.572). This congruency effect reflected higher accuracy on congruent trials (M = .941, SD = .049) compared with incongruent trials (M = .905, SD = .066). Furthermore, there was evidence for excluding the interaction between memory load and congruency (BF₍excl₎ = 2.848). Accuracy proportions across all conditions are shown in Fig S1.2.

**Table S1.2. Descriptive Statistics of accuracy (proportion correct) in the spatial conflict task (N=97).**

| Condition | Mean | Std. Deviation | Minimum | Maximum |
| --- | --- | --- | --- | --- |
| Low memory and congruent | .951 | .051 | .758 | 1 |
| Low memory and incongruent | .909 | .075 | .658 | 1 |
| High memory and congruent | .931 | .064 | .657 | 1 |
| High memory and incongruent | .902 | .085 | .537 | 1 |

**Fig S1.2. Accuracy (proportion correct) in the spatial conflict task.** Error bars indicate ±1 standard error of the mean (SEM).
