# Supplementary material for: Concurrent measurement of working memory and inhibitory control and their correlations with autistic and ADHD traits in the general population
Source: PLoS One. 2026 Jan 5;21(1):e0339846. doi: 10.1371/journal.pone.0339846 (PMC12768290; doi:10.1371/journal.pone.0339846)
Supplement: S2 Appendix — (DOCX) [file pone.0339846.s002.docx]

**S2 Appendix: Speed–accuracy trade-off in Study 1**

Although the patterns of RT and accuracy were not opposing, this analysis was conducted to evaluate potential speed–accuracy trade-offs.

To account for such trade-offs, inverse efficiency (IE = RT / proportion of correct responses) scores were calculated for each participant. This approach allows performance efficiency to be evaluated while considering possible compensatory strategies.

Results of the inverse efficiency analyses for Study 1 are presented below, first for the flanker task and then for the spatial conflict task.

**S2a) Inverse Efficiency – Speed–Accuracy Trade-off (Flanker Task)**

A Bayesian repeated-measures ANOVA on inverse efficiency showed that the best-fitting model included the main effects of memory load and congruency (BF₁₀ = 5.41 × 10²⁸ against the null model). There was strong evidence for including both memory load (BF₍incl₎ = 8.90 × 10¹⁹) and congruency (BF₍incl₎ = 6.68 × 10⁸), and moderate evidence against including the interaction (BF₍excl₎ = 4.300). This pattern was consistent with the RT results (see related RT results for the flanker task in Study 1 in the main body) and was not opposed by the accuracy results (see S1 Appendix). The convergence across measures, with no opposing trends between RT and accuracy, indicates that participants did not trade speed for accuracy in the flanker task. Fig S2.1 shows inverse efficiency values (measured in milliseconds [ms]) across memory load and congruency conditions in the flanker task.

**Fig S2.1. Inverse efficiency (ms) in the flanker task.** Error bars indicate ±1 standard error of the mean (SEM).





**S2b) Inverse Efficiency – Speed–Accuracy Trade-off (Spatial conflict Task)**

A Bayesian repeated-measures ANOVA on inverse efficiency showed that the best-fitting model included only the main effect of congruency (BF₁₀ = 5.21 × 10^13^ against the null model). The analysis provided strong evidence for including a congruency effect (BF₍incl₎ = 5.04 × 10¹³), moderate evidence *against* including a memory-load effect (BF₍excl₎ = 4.43), and weak evidence for including the memory × congruency interaction (BF₍incl₎ = 2.87). Fig S2.2 shows inverse efficiency values (in milliseconds) across memory-load and congruency conditions in the spatial conflict task. This outcome aligns with the RT findings reported in the main body for the spatial conflict task and is not in opposition to the accuracy results presented in S1 Appendix. The consistent pattern across these measures suggests that participants did not engage in a speed–accuracy trade-off in the spatial conflict task.

**Fig S2.2. Inverse efficiency (ms) in the spatial conflict task.** Error bars indicate ±1 standard error of the mean (SEM).
