# Supplementary material for: Concurrent measurement of working memory and inhibitory control and their correlations with autistic and ADHD traits in the general population
Source: PLoS One. 2026 Jan 5;21(1):e0339846. doi: 10.1371/journal.pone.0339846 (PMC12768290; doi:10.1371/journal.pone.0339846)
Supplement: S3 Appendix — (DOCX) [file pone.0339846.s003.docx]

**S3 Appendix: Correlations between the measures from the cognitive tasks and ASC traits (Study 1)**

**S3a) Descriptive Statistics of ASC traits in study 1**

Descriptive statistics of ASC traits, measured by the Short Autism-Spectrum Quotient (AQ-S), are presented in Table S3.1. The AQ-S includes two subscales: Social Behaviour and Numbers and Patterns. Table 1 presents the descriptive statistics for the total AQ score and each subscale.

**Table S3.1. Descriptive statistics of ASC traits (N=93).**

| Measure | Mean | Std. Deviation | Minimum | Maximum |
| --- | --- | --- | --- | --- |
| AQ-S total score | 62.23 | 11.41 | 42.0 | 98.0 |
| Social behaviour subscale score | 50.77 | 10.17 | 32.0 | 78.0 |
| Number and pattern subscale score | 11.45 | 3.279 | 5.0 | 20.0 |

**S3b) Bayesian analysis results for the correlations between the measures from the cognitive tasks and ASC traits in Study 1**

The results of the Bayesian Pearson correlations between each task performance measure and AQ-S scores are presented below. Results are shown first for the flanker task (reaction time, accuracy, and inverse efficiency, respectively) and then for the spatial conflict task (reaction time, accuracy, and inverse efficiency, respectively).

**Flanker task**

**S3b1) Bayesian analysis results for the correlations between reaction time in the flanker task and ASC traits.**

Table S3.2 presents the Bayesian correlations between log-transformed reaction times in the flanker task and AQ-S total and subscale scores, including the posterior mean correlations, 95% credible intervals, and Bayes Factors.

**Table S3.2. Bayesian correlations between log-transformed reaction times of flanker task and AQ-S total and subscale scores.**

| Condition | AQ subscale | r | BF₀₁ | 95% CI for r |
| --- | --- | --- | --- | --- |
| Low & congruent | AQ total score | 0.056 | 6.708 | [-0.147, 0.253] |
| Low & congruent | Social behaviour score | 0.065 | 6.377 | [-0.138, 0.262] |
| Low & congruent | Number and pattern score | -0.008 | 7.695 | [-0.208, 0.193] |
| Low & incongruent | AQ_total score | 0.017 | 7.619 | [-0.185, 0.216] |
| Low & incongruent | Social behaviour score | 0.032 | 7.365 | [-0.170, 0.231] |
| Low & incongruent | Number and pattern score | -0.042 | 7.137 | [-0.240, 0.161] |
| High & congruent | AQ total score | 0.160 | 2.433 | [-0.045, 0.347] |
| High & congruent | Social behaviour score | 0.136 | 3.358 | [-0.069, 0.326] |
| High & congruent | Number and pattern score | 0.135 | 3.407 | [-0.070, 0.325] |
| High & incongruent | AQ total score | 0.170 | 2.100 | [-0.035, 0.356] |
| High&incongruent | Social behaviour score | 0.155 | 2.595 | [-0.049, 0.343] |
| High&incongruent | Number and pattern score | 0.109 | 4.548 | [-0.096, 0.301] |

Note. Bayes Factors are BF_01_ values showing the evidence *against* an association

Furthermore, Fig S3.1 shows the Bayesian Pearson correlation matrix between mean reaction time in each flanker condition and AQ-S total score. The vertical line represents the AQ-S diagnostic cut-off score of 65, included following reviewer feedback to help contextualize autistic trait levels relative to the diagnostic range. Although this study focuses on autistic traits within the general population and adopts a transdiagnostic perspective, the line might provide a useful reference point for understanding the distribution of scores.

**Fig S3.1. Bayesian Pearson correlation matrix between reaction time (measured in milliseconds) in the flanker task and AQ-S total score (vertical line indicates AQ-S diagnostic cut-off score of 65).**

**
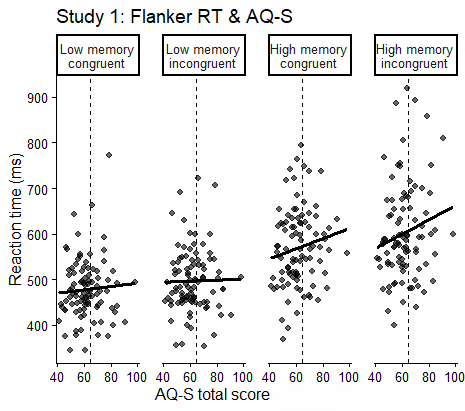
**

**S3b2) Bayesian analysis results for the correlations between accuracy in the flanker task and ASC traits.**

The results of the Bayesian Pearson correlations between accuracy proportions in the flanker task and AQ-S total and subscale scores are presented in Table S3.3, including the posterior mean correlations, 95% credible intervals, and Bayes Factors.

**Table S3.3. Bayesian correlations between accuracy proportions of flanker task and AQ-S total and subscale scores.**

| Condition | AQ subscale | r | BF₀₁ | 95% CI for r |
| --- | --- | --- | --- | --- |
| Low & congruent | AQ total score | -0.002 | 7.713 | [-0.203, 0.198] |
| Low & congruent | Social behaviour score | 0.022 | 7.557 | [-0.180, 0.221] |
| Low & congruent | Number and pattern score | -0.076 | 5.980 | [-0.271, 0.128] |
| Low & incongruent | AQ total score | -0.063 | 6.463 | [-0.259, 0.140] |
| Low & incongruent | Social behaviour score | -0.044 | 7.080 | [-0.242, 0.159] |
| Low & incongruent | Number and pattern score | -0.083 | 5.666 | [-0.278, 0.121] |
| High & congruent | AQ total score | 0.075 | 6.000 | [-0.129, 0.270] |
| High & congruent | Social behaviour score | 0.083 | 5.674 | [-0.121, 0.278] |
| High & congruent | Number and pattern score | 0.004 | 7.710 | [-0.197, 0.204] |
| High & incongruent | AQ total score | -0.064 | 6.424 | [-0.260, 0.139] |
| High & incongruent | Social behaviour score | -0.028 | 7.450 | [-0.227, 0.174] |
| High & incongruent | Number and pattern score | -0.136 | 3.356 | [-0.326, 0.069] |

Note. Bayes Factors are BF_01_ values showing the evidence *against* an association

Additionally, Fig S3.2 presents the Bayesian Pearson correlation matrix between accuracy proportions in each flanker condition and AQ-S total score.

**Fig S3.2. Bayesian Pearson correlation matrix between accuracy proportions in each flanker condition and AQ-S total score (vertical line indicates AQ-S diagnostic cut-off score of 65).**

**
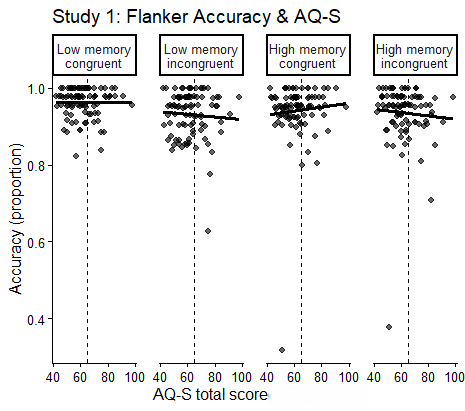
**

**S3b3) Bayesian analysis results for the correlations between inverse efficiency in the flanker task and ASC traits.**

Table S3.4 represents the Bayesian correlations between inverse efficiency scores (IES) in the flanker task and AQ-S total and subscale scores, reporting the posterior mean correlations, 95% credible intervals, and Bayes Factors.

**Table S3.4. Bayesian correlations between inverse efficiency scores of the flanker task and AQ-S total and subscale scores**

| Condition | AQ subscale | r | BF₀₁ | 95% CI for r |
| --- | --- | --- | --- | --- |
| Low & congruent | AQ total score | 0.055 | 6.751 | [−0.148, 0.252] |
| Low & congruent | Social behaviour score | 0.057 | 6.684 | [−0.147, 0.254] |
| Low & congruent | Number and pattern score | 0.015 | 7.643 | [−0.187, 0.214] |
| Low & incongruent | AQ total score | 0.047 | 7.000 | [−0.156, 0.244] |
| Low & incongruent | Social behaviour score | 0.051 | 6.878 | [−0.152, 0.248] |
| Low & incongruent | Number and pattern score | 0.005 | 7.706 | [−0.196, 0.205] |
| High & congruent | AQ total score | 0.080 | 5.787 | [−0.124, 0.275] |
| High & congruent | Social behaviour score | 0.061 | 6.518 | [−0.142, 0.258] |
| High & congruent | Number and pattern score | 0.089 | 5.429 | [−0.116, 0.283] |
| High & incongruent | AQ total score | 0.160 | 2.417 | [−0.044, 0.348] |
| High & incongruent | Social behaviour score | 0.136 | 3.371 | [−0.069, 0.325] |
| High & incongruent | Number and pattern score | 0.137 | 3.302 | [−0.067, 0.327] |

Note. Bayes Factors are BF_01_ values showing the evidence *against* an association

Furthermore, the Bayesian Pearson correlation matrix between inverse efficiency scores in each flanker condition and AQ-S total score is shown in Fig S3.3.

**Fig S3.3. Bayesian Pearson correlation matrix between inverse efficiency scores in each flanker condition and AQ-S total score (vertical line indicates AQ-S diagnostic cut-off score of 65).**

**
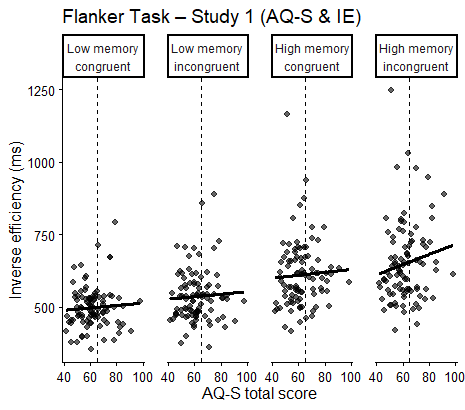
**

**Spatial conflict task**

**S3b4) Bayesian analysis results for the correlations between reaction time in the spatial conflict task and ASC traits.**

Table S3.5 presents the Bayesian Pearson correlations between log-transformed reaction times in the spatial conflict task and AQ-S total and subscale scores which includes the posterior mean correlations, 95% credible intervals, and Bayes Factors.

**Table S3.5. Bayesian correlations between log-transformed reaction times of the spatial conflict task and AQ-S total and subscale score.**

| Condition | AQ subscale | r | BF₀₁ | 95% CI for r |
| --- | --- | --- | --- | --- |
| Low & congruent | AQ total score | 0.044 | 7.099 | [−0.157, 0.241] |
| Low & congruent | Social behaviour score | 0.066 | 6.378 | [−0.137, 0.261] |
| Low & congruent | Number and pattern score | −0.050 | 6.924 | [−0.247, 0.152] |
| Low & incongruent | AQ total score | 0.106 | 4.649 | [−0.097, 0.298] |
| Low & incongruent | Social behaviour score | 0.117 | 4.161 | [−0.086, 0.308] |
| Low & incongruent | Number and pattern score | 0.006 | 7.742 | [−0.194, 0.206] |
| High & congruent | AQ total score | 0.054 | 6.787 | [−0.148, 0.250] |
| High & congruent | Social behaviour score | 0.091 | 5.331 | [−0.112, 0.284] |
| High & congruent | Number and pattern score | −0.093 | 5.223 | [−0.286, 0.110] |
| High & incongruent | AQ total score | 0.133 | 3.489 | [−0.071, 0.322] |
| High & incongruent | Social behaviour score | 0.156 | 2.572 | [−0.048, 0.342] |
| High & incongruent | Number and pattern score | −0.021 | 7.598 | [−0.220, 0.179] |

Note. Bayes Factors are BF_01_ values showing the evidence *against* an association

The Bayesian Pearson correlation matrix in Fig S3.4 illustrates the associations between reaction times across the spatial conflict task conditions and the AQ-S total score.

**Fig S3.4. Bayesian Pearson correlation matrix between reaction times in each spatial conflict condition and AQ-S total score (vertical line indicates AQ-S diagnostic cut-off score of 65).**


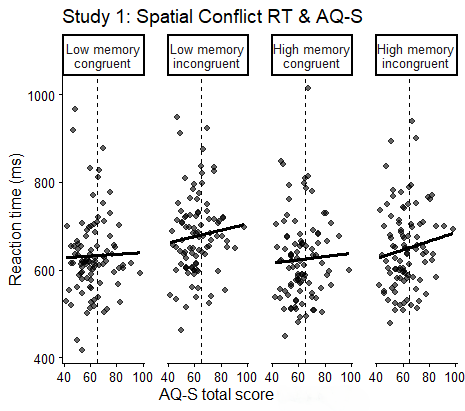


**S3b5) Bayesian analysis results for the correlations between accuracy in the spatial conflict task and ASC traits.**

Table S3.6 summarises the Bayesian Pearson correlations between accuracy proportions in the spatial conflict task and AQ-S total and subscale scores, including the posterior mean correlations, 95% credible intervals, and Bayes Factors.

**Table S3.6. Bayesian correlations between the spatial conflict task accuracy (proportion correct) and AQ-S total and subscale scores.**

| Condition | AQ subscale | r | BF₀₁ | 95% CI for r |
| --- | --- | --- | --- | --- |
| Low & congruent | AQ total score | -0.194 | 1.383 | [-0.376, 0.009] |
| Low & congruent | Social behaviour score | -0.172 | 2.018 | [-0.357, 0.032] |
| Low & congruent | Number and pattern score | -0.142 | 3.087 | [-0.330, 0.061] |
| Low & incongruent | AQ total score | -0.072 | 6.121 | [-0.267, 0.130] |
| Low & incongruent | Social behaviour score | -0.035 | 7.339 | [-0.232, 0.166] |
| Low & incongruent | Number and pattern score | -0.144 | 3.032 | [-0.332, 0.060] |
| High & congruent | AQ total score | 0.082 | 5.722 | [-0.121, 0.276] |
| High & congruent | Social behaviour score | 0.100 | 4.941 | [-0.103, 0.292] |
| High & congruent | Number and pattern score | -0.024 | 7.553 | [-0.222, 0.177] |
| High & incongruent | AQ total score | -0.117 | 4.187 | [-0.307, 0.087] |
| High & incongruent | Social behaviour score | -0.075 | 6.007 | [-0.270, 0.128] |
| High & incongruent | Number and pattern score | -0.173 | 1.983 | [-0.358, 0.031] |

Note. Bayes Factors are BF_01_ values showing the evidence *against* an association

Furthermore, the Bayesian Pearson correlation matrix in Fig S3.5 depicts the correlations between accuracy proportions across the spatial conflict task conditions and the AQ-S total score.

**Fig S3.5. Bayesian Pearson correlation matrix between accuracy proportions in each spatial conflict condition and AQ-S total score (vertical line indicates AQ-S diagnostic cut-off score of 65).**

**
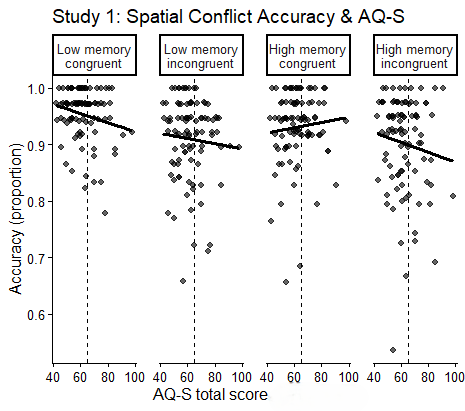
**

**S3b6) Bayesian analysis results for the correlations between inverse efficiency in the spatial conflict task and ASC traits.**

Table S3.7 reports the Bayesian correlations between inverse efficiency scores in the spatial conflict task and AQ-S total and subscale scores, presenting the posterior mean correlations, 95% credible intervals, and Bayes Factors.

**Table S3.7. Bayesian correlations between log-transformed inverse efficiency scores of spatial conflict task and AQ-S total and subscale scores**

| Condition | AQ subscale | r | BF₀₁ | 95% CI for r |
| --- | --- | --- | --- | --- |
| Low & congruent | AQ total score | 0.106 | 4.656 | [-0.097, 0.298] |
| Low & congruent | Social behaviour score | 0.118 | 4.131 | [-0.086, 0.308] |
| Low & congruent | Number and pattern score | 0.004 | 7.752 | [-0.196, 0.203] |
| Low & incongruent | AQ total score | 0.125 | 3.796 | [-0.078, 0.315] |
| Low & incongruent | Social behaviour score | 0.115 | 4.245 | [-0.088, 0.306] |
| Low & incongruent | Number and pattern score | 0.079 | 5.852 | [-0.124, 0.273] |
| High & congruent | AQ total score | 0.011 | 7.718 | [-0.190, 0.209] |
| High & congruent | Social behaviour score | 0.033 | 7.387 | [-0.168, 0.230] |
| High & congruent | Number and pattern score | -0.066 | 6.384 | [-0.261, 0.137] |
| High & incongruent | AQ total score | 0.171 | 2.039 | [-0.033, 0.356] |
| High & incongruent | Social behaviour score | 0.165 | 2.238 | [-0.039, 0.351] |
| High & incongruent | Number and pattern score | 0.083 | 5.675 | [-0.120, 0.277] |

Note. Bayes Factors are BF_01_ values showing the evidence *against* an association

The Bayesian Pearson correlation matrix in Fig S3.6 depicts the correlation between inverse efficiency scores across spatial conflict task conditions and the AQ-S total score.

**Fig S3.6. Bayesian Pearson correlation matrix between inverse efficiency scores (IES) in each spatial conflict condition and AQ-S total score (vertical line indicates AQ-S diagnostic cut-off score of 65).**

**
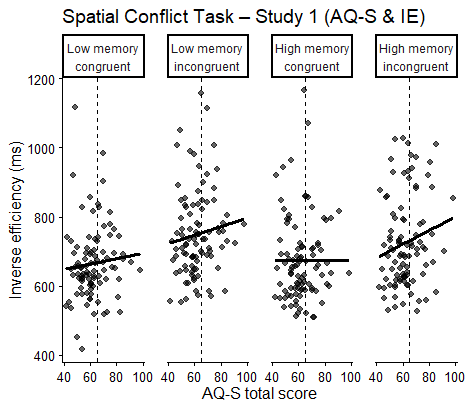
**
