# Supplementary material for: Concurrent measurement of working memory and inhibitory control and their correlations with autistic and ADHD traits in the general population
Source: PLoS One. 2026 Jan 5;21(1):e0339846. doi: 10.1371/journal.pone.0339846 (PMC12768290; doi:10.1371/journal.pone.0339846)
Supplement: S4 Appendix — (DOCX) [file pone.0339846.s004.docx]

**S4 Appendix: Partial correlations between cognitive task measures and ASC traits (Study 1)**

Although the preregistration focused on associations between the task measures and the AQ-S total score, this supplementary appendix provides additional analyses for the two AQ-S subscales in response to reviewer feedback. These revealed no evidence of a meaningful correlation between the size of any congruency or memory effects and ASC traits on any AQ-S subscale.

**S4a) Partial correlations between the flanker task measures and ASC traits.**

Table S4.1 presents the results of the Bayesian regression analyses (equivalent to partial correlations) examining associations between the two AQ-S subscales (Social Behaviour and Numbers & Patterns) and performance on the flanker task. It includes results for correlations between incongruent-trial performance and each AQ-S subscale (controlling for congruent trials) and between high-memory performance and each AQ-S subscale (controlling for low-memory trials), reported separately for RT, accuracy, and inverse efficiency.

**Table S4.1. Partial correlations between AQ-S subscales and the flanker task performance.**

| AQ-S Subscale | Partial correlation design | RT | Accuracy | Inverse efficiency |
| --- | --- | --- | --- | --- |
| Social Behaviour | Incongruent trial performance (controlling for congruent) | BF₍incl₎=0.025  Mean=1.263×10⁻⁶  95% CI = [0.000, 0.000] | BF₍incl₎=0.186  Mean=1.024×10⁻⁴  95% CI = [−8.742×10⁻⁴, 0.002] | BF₍incl₎=0.050  Mean=−4.719×10⁻⁶  95% CI = [0.000, 0.000] |
|  | High memory performance (controlling for low memory) | BF₍incl₎=0.294  Mean=−4.571×10⁻⁴  95% CI = [−0.007, 0.003] | BF₍incl₎=0.228  Mean=1.480×10⁻⁴  95% CI = [−7.562×10⁻⁴, 0.002] | BF₍incl₎=0.172  Mean=−3.391×10⁻⁴  95% CI = [−0.004, 0.004] |
| Numbers & Patterns | Incongruent trial performance (controlling for congruent) | BF₍incl₎=0.025  Mean=−5.210×10⁻⁶  95% CI = [0.000, 0.000] | BF₍incl₎ =0.266  Mean=−2.825×10⁻⁴  95% CI = [−0.003, 6.066×10⁻⁵] | BF₍incl₎=0.056  Mean=8.981×10⁻⁵  95% CI = [0.000, 0.000] |
|  | High memory performance (controlling for low memory) | BF₍incl₎=0.393  Mean=0.001  95% CI = [0.000, 0.010] | BF₍incl₎=0.234  Mean=−1.184×10⁻⁴  95% CI = [−0.003, 6.069×10⁻⁵] | BF₍incl₎=0.239  Mean=7.625×10⁻⁴  95% CI = [−1.156×10⁻⁴, 0.008] |

Note, BF₍inclusion₎ is the Bayes factor comparing models that include a predictor against models that exclude it.

**S4b) Partial correlations between the spatial conflict task measures and ASC traits**

Table S4.2 summarises the Bayesian regression analyses (serving as partial correlations) examining how the two AQ-S subscales—Social Behaviour and Numbers & Patterns—relate to performance on the spatial conflict task. It presents the associations between the AQ subscales and both incongruent-trial performance (adjusted for congruent trials) and high-memory performance (adjusted for low-memory trials), shown separately for RT, accuracy, and inverse efficiency.

**Table S4.2. Partial correlations between AQ-S subscales and the spatial conflict task performance.**

| AQ-S Subscale | Partial correlation design | RT | Accuracy | Inverse efficiency |
| --- | --- | --- | --- | --- |
| Social Behaviour | Incongruent trial performance (controlling for congruent) | BF₍incl₎=0.110  Mean=−2.516×10⁻⁵  95% CI = [0.000, 0.001] | BF₍incl₎=0.205  Mean=2.983×10⁻⁴  95% CI = [−0.001, 0.003] | BF₍incl₎=0.239  Mean=−3.830×10⁻⁴  95% CI = [−0.005, 0.002] |
|  | High memory performance (controlling for low memory) | BF₍incl₎=0.143  Mean=2.824×10⁻⁴  95% CI = [−4.507×10⁻⁴, 0.003] | BF₍incl₎=0.161  Mean=1.207×10⁻⁴  95% CI = [−7.498×10⁻⁴, 0.001] | BF₍incl₎=0.142  Mean=1.455×10⁻⁴  95% CI = [−9.154×10⁻⁴, 0.002] |
| Numbers & Patterns | Incongruent trial performance (controlling for congruent) | BF₍incl₎=0.149  Mean=2.516×10⁻⁴  95% CI = [0.000, 0.003] | BF₍incl₎=0.414  Mean=−5.369×10⁻⁴  95% CI = [−0.004, 2.731×10⁻⁴] | BF₍incl₎=0.409  Mean=0.001  95% CI = [0.000, 0.008] |
|  | High memory performance (controlling for low memory) | BF₍incl₎=0.132  Mean=−1.835×10⁻⁴  95% CI = [−0.003, 0.000] | BF₍incl₎=0.159  Mean=−9.060×10⁻⁵  95% CI = [−0.002, 2.945×10⁻⁴] | BF₍incl₎=0.134  Mean=−6.840×10⁻⁵  95% CI = [−0.002, 5.871×10⁻⁴] |

Note, BF₍inclusion₎ is the Bayes factor comparing models that include a predictor against models that exclude it.
