# Supplementary material for: Concurrent measurement of working memory and inhibitory control and their correlations with autistic and ADHD traits in the general population
Source: PLoS One. 2026 Jan 5;21(1):e0339846. doi: 10.1371/journal.pone.0339846 (PMC12768290; doi:10.1371/journal.pone.0339846)
Supplement: S5 Appendix — (DOCX) [file pone.0339846.s005.docx]

**S5 Appendix: Partial correlations between cognitive task measures and ASC traits with age and gender as covariates (Study 1)**

Although these analyses were not preregistered, this supplementary appendix includes additional Bayesian regression analyses (equivalent to partial correlations) examining the associations between ASC traits and task performance with age and gender included as covariates. These analyses showed no meaningful correlations between the size of any congruency or memory effects and ASC traits across the AQ-S subscales after adjusting for these covariates (see Tables S5.1 and S5.2 below).

**S5a) Partial correlations between the flanker task measures and ASC traits (with age & gender as covariates).**

Table S5.1 presents the results of the Bayesian regression analyses (equivalent to partial correlations) examining associations between AQ-S scores and performance on the flanker task. The table reports correlations between incongruent-trial performance and AQ-S scores (controlling for congruent trials), and between high-memory performance and AQ-S scores (controlling for low-memory trials), with age and gender included as covariates. Results are shown separately for RT, accuracy, and inverse efficiency.

**Table S5.1. Partial correlations between AQ-S and the flanker task performance (with age & gender as covariates).**

| AQ-S Subscale | Partial correlation design | RT | Accuracy | Inverse efficiency |
| --- | --- | --- | --- | --- |
| Total score | Incongruent trial performance (controlling for congruent trials, age, gender) | BF₍incl₎=0.039 Mean=3.391×10⁻⁶ 95%CI=[0.000,0.000] | BF₍incl₎=0.350 Mean=-1.458×10⁻⁴  95%CI=[-9.022×10⁻⁴,2.287×10⁻⁴] | BF₍incl₎=0.105 Mean=7.658×10⁻⁵ 95%CI=[-1.512×10⁻⁴,0.001] |
|  | High-memory performance (controlling for low memory, age, gender) | BF₍incl₎=0.812 Mean=9.939×10⁻⁴ 95%CI=[-0.001,0.005] | BF₍incl₎=0.350 Mean=1.603×10⁻⁵ 95%CI=[-7.788×10⁻⁴,0.001] | BF₍incl₎=0.357 Mean=4.439×10⁻⁴ 95%CI=[-6.177×10⁻⁴,0.003] |
| Social Behaviour | Incongruent trial performance (controlling for congruent trials, age, gender) | BF₍incl₎=0.039  Mean=3.467×10⁻⁶  95%CI=[0.000,0.000] | BF₍incl₎=0.296  Mean=1.331×10⁻⁵  95%CI=[−7.538×10⁻⁴,6.105×10⁻⁶] | BF₍incl₎=0.094  Mean=2.199×10⁻⁵  95%CI=[−2.558×10⁻⁶,6.000×10⁻⁴] |
|  | High-memory performance (controlling for low memory, age, gender) | BF₍incl₎=0.600  Mean=2.333×10⁻⁵  95%CI=[−0.002,0.004] | BF₍incl₎=0.357  Mean=8.931×10⁻⁵  95%CI=[−4.817×10⁻⁴,0.001] | BF₍incl₎=0.312  Mean=−7.249×10⁻⁵  95%CI=[−4.884×10⁻⁴,0.003] |
| Numbers & Patterns | Incongruent trial performance (controlling for congruent trials, age, gender) | BF₍incl₎=0.039  Mean=−6.322×10⁻⁸  95%CI=[0.000,0.000] | BF₍incl₎=0.404  Mean=−5.005×10⁻⁴  95%CI=[−0.003,2.584×10⁻⁴] | BF₍incl₎=0.103  Mean=2.043×10⁻⁴  95%CI=[−0.001,0.002] |
|  | High-memory performance (controlling for low memory, age, gender) | BF₍incl₎=0.678  Mean=0.002  95%CI=[−0.002,0.011] | BF₍incl₎=0.362  Mean=−3.469×10⁻⁴  95% CI=[−0.004,0.001] | BF₍incl₎=0.427  Mean=0.002  95%CI=[−2.406×10⁻⁴,0.011] |

Note, BF₍inclusion₎ is the Bayes factor comparing models that include a predictor against models that exclude it. Values below 1 indicate that the data favour the model without the predictor.

**S5b) Partial correlations between the spatial conflict task measures and ASC traits (with age & gender as covariates).**

Table S5.2 summarises the correlations between AQ-S scores and performance on the spatial conflict task. It includes associations for incongruent-trial performance (controlling for congruent trials) and for high-memory performance (controlling for low-memory trials), with age and gender entered as covariates. Results are presented separately for RT, accuracy, and inverse efficiency.

**Table S5.2. Partial correlations between AQ-S subscales and spatial conflict task performance (with age & gender as covariates).**

| AQ-S Subscale | Partial correlation design | RT | Accuracy | Inverse efficiency |
| --- | --- | --- | --- | --- |
| Total score | Incongruent-trial performance (controlling for congruent trials, age, gender) | BF₍incl₎=0.309 Mean=2.232×10⁻⁴ 95%CI=[0.000,0.001] | BF₍incl₎=0.376 Mean=-1.316×10⁻⁴ 95%CI=[-0.001,0.001] | BF₍incl₎=0.409 Mean=4.345×10⁻⁴ 95%CI=[-7.263×10⁻⁴,0.003] |
|  | High-memory performance (controlling for low memory, age, gender) | BF₍incl₎=0.187 Mean=5.113×10⁻⁵  95%CI=[-3.474×10⁻⁴,0.002] | BF₍incl₎=0.276 Mean=3.831×10⁻⁵ 95%CI=[-2.978×10⁻⁴,0.001] | BF₍incl₎=0.194 Mean=3.697×10⁻⁵ 95%CI=[-9.959×10⁻⁴,0.002] |
| Social Behaviour | Incongruent-trial performance (controlling for congruent trials, age, gender) | BF₍incl₎=0.203  Mean=3.328×10⁻⁵  95%CI=[0.000,0.001] | BF₍incl₎=0.354  Mean=1.086×10⁻⁴  95%CI=[−9.956×10⁻⁴,6.320×10⁻⁴] | BF₍incl₎=0.327  Mean=−1.067×10⁻⁴  95%CI=[−2.620×10⁻⁴,0.002] |
|  | High-memory performance (controlling for low memory, age, gender) | BF₍incl₎=0.197  Mean=1.297×10⁻⁴  95%CI=[−2.327×10⁻⁴,0.002] | BF₍incl₎=0.294  Mean=1.017×10⁻⁴  95%CI=[−2.955×10⁻⁴,9.697×10⁻⁴] | BF₍incl₎=0.195  Mean=4.493×10⁻⁵  95%CI=[−7.897×10⁻⁴,0.001] |
| Numbers & Patterns | Incongruent-trial performance (controlling for congruent trials, age, gender) | BF₍incl₎=0.252  Mean=4.825×10⁻⁴  95%CI=[0.000,0.004] | BF₍incl₎=0.585  Mean=−9.877×10⁻⁴  95%CI=[−0.006,0.000] | BF₍incl₎=0.649  Mean=0.002  95%CI=[−8.313×10⁻⁵,0.009] |
|  | High-memory performance (controlling for low memory, age, gender) | BF₍incl₎=0.189  Mean=−4.014×10⁻⁴  95%CI=[−0.007,4.502×10⁻⁴] | BF₍incl₎=0.279  Mean=−3.226×10⁻⁴  95%CI=[−0.004,7.928×10⁻⁴] | BF₍incl₎=0.192  Mean=−8.123×10⁻⁵  95%CI=[−0.005,0.004] |

Note, BF₍inclusion₎ is the Bayes factor comparing models that include a predictor against models that exclude it. Values below 1 indicate that the data favour the model without the predictor.
