# Supplementary material for: Concurrent measurement of working memory and inhibitory control and their correlations with autistic and ADHD traits in the general population
Source: PLoS One. 2026 Jan 5;21(1):e0339846. doi: 10.1371/journal.pone.0339846 (PMC12768290; doi:10.1371/journal.pone.0339846)
Supplement: S6 Appendix — (DOCX) [file pone.0339846.s006.docx]

**S6 Appendix: Accuracy in the flanker task and spatial conflict task in Study 2**

**S6a) Accuracy in the flanker task**

Table S6.1 displays the descriptive statistics for participants’ accuracy (proportion correct) in the flanker task.

**Table S6.1. Descriptive Statistics of accuracy (proportion correct) in the flanker task (N=97).**

| Condition | Mean | Std. Deviation | Minimum | Maximum |
| --- | --- | --- | --- | --- |
| Low memory and congruent | .967 | .043 | .736 | 1 |
| Low memory and incongruent | .964 | .041 | .817 | 1 |
| High memory and congruent | .937 | .094 | .405 | 1 |
| High memory and incongruent | .934 | .095 | .359 | 1 |

A Bayesian repeated-measures ANOVA on accuracy showed that the best-fitting model included only the main effect of memory load (BF₀₁ = 0.013 relative to the null model). There was strong evidence for including the memory effect (BF₍incl₎ = 75.241), with participants being more accurate in low-memory trials (M = .960, SD = .064) than in high-memory trials (M = .935, SD = .093). There was moderate evidence for excluding the main effect of congruency (BF₍excl₎ = 5.014) and, likewise, moderate evidence for excluding the interaction between memory load and congruency (BF₍excl₎ = 6.286). Accuracy proportions across memory and congruency conditions in the flanker task are shown in Fig S6.1.

**Fig S6.1. Accuracy (proportion correct) in the flanker task.** Error bars indicate ±1 standard error of the mean (SEM).





**S6b) Accuracy in the spatial conflict task**

Table S6.2 displays the descriptive statistics for participants’ accuracy in the spatial conflict task.

**Table S6.2. Descriptive statistics of accuracy (proportion correct) in the spatial conflict task (N=94).**

| Condition | Mean | Std. Deviation | Minimum | Maximum |
| --- | --- | --- | --- | --- |
| Low memory and congruent | .969 | .042 | .745 | 1 |
| Low memory and incongruent | .923 | .066 | .700 | 1 |
| High memory and congruent | .948 | .064 | .563 | 1 |
| High memory and incongruent | .933 | .074 | .535 | 1 |

A Bayesian repeated-measures ANOVA on accuracy data showed that the best-fitting model included memory load, congruency, and their interaction (BF₁₀ = 2.71 × 10³ relative to the null model). There was weak evidence for excluding the main effect of memory load (BF₍excl₎ = 2.926). In contrast, there was very strong evidence for including the main effect of congruency (BF₍incl₎ = 8.24 × 10⁷), with participants being more accurate on congruent trials (M = .958, SD = .044) than on incongruent trials (M = .928, SD = .061). There was also strong evidence for including the interaction between memory load and congruency (BF₍incl₎ = 2661.102). Follow-up Bayesian tests showed strong evidence for a memory effect within congruent trials (BF₁₀ = 18.68), but moderate evidence for the absence of a memory effect within incongruent trials (BF₀₁ = 2.62). Furthermore, there was strong evidence for a congruency effect in the high-memory condition (BF₁₀ = 12.77) and even stronger evidence for this effect in the low-memory condition (BF₁₀ = 4.197 × 10⁹). Fig S6.2 displays accuracy proportions across memory load and congruency conditions in the spatial conflict task.

**Fig S6.2. Accuracy (proportion correct) in the spatial conflict task**. Error bars indicate ±1 standard error of the mean (SEM).
