# Supplementary material for: Concurrent measurement of working memory and inhibitory control and their correlations with autistic and ADHD traits in the general population
Source: PLoS One. 2026 Jan 5;21(1):e0339846. doi: 10.1371/journal.pone.0339846 (PMC12768290; doi:10.1371/journal.pone.0339846)
Supplement: S7 Appendix — (DOCX) [file pone.0339846.s007.docx]

**S7 Appendix: Speed–accuracy trade-off in Study 2**

Similar to Study 1, although the patterns of RT and accuracy were not opposing, this analysis was conducted to evaluate potential speed–accuracy trade-offs. To account for such effects, inverse efficiency (IE = RT / proportion of correct responses) scores were calculated for each participant. This measure reflects performance efficiency while considering possible compensatory strategies.

Results of the trade-off analysis for Study 2 are presented below, first for the flanker task and then for the spatial conflict task.

**S7a) Inverse Efficiency – Speed–Accuracy Trade-off (Flanker Task)**

A Bayesian repeated-measures ANOVA on inverse efficiency (IE) scores showed that the best-fitting model included memory load, congruency, and their interaction (BF₁₀ = 6.94 × 10⁻²⁴ relative to the null model). There was strong evidence for including the main effect of memory load (BF₍incl₎ = 7.95 × 10²¹). In contrast, there was moderate evidence for excluding the main effect of congruency (BF₍excl₎ = 4.81). There was also strong evidence for including the interaction between memory load and congruency (BF₍incl₎ = 85.82). This pattern was consistent with the RT results (see results for the flanker task in Study 2 in the main body) and was not in opposition to the accuracy results (see S6 Appendix). The consistency across measures, with no opposing trends between RT and accuracy, indicates that participants did not trade speed for accuracy in the flanker task. Fig S7.1 shows inverse efficiency values (measured in milliseconds [ms]) across memory load and congruency conditions in the flanker task.

**Fig S7.1. Inverse efficiency (ms) in the flanker task.** Error bars indicate ±1 standard error of the mean (SEM).





**S7b) Inverse Efficiency – Speed–Accuracy Trade-off (Spatial Conflict Task)**

A Bayesian repeated-measures ANOVA on inverse efficiency (IE) scores showed that the best-fitting model included memory load, congruency, and their interaction (BF₁₀ = 6.66 × 10²³ relative to the null model). There was strong evidence for including the main effects of memory load (BF₍incl₎ = 1.28 × 10⁹) and congruency (BF₍incl₎ = 2.87 × 10¹⁰), and strong evidence for including the memory × congruency interaction (BF₍incl₎ = 17527). This pattern was consistent with the RT results (presented in the results section of Study 2 in the main body) and was not in opposition to the accuracy results (see S6 Appendix). The consistent pattern across measures, with no opposing trends between RT and accuracy, indicates that participants did not trade speed for accuracy in the spatial conflict task. Fig S7.2 shows inverse efficiency (measured in milliseconds [ms]) values across memory load and congruency conditions in the spatial conflict task.

**Fig S7.2. Inverse efficiency (ms) in the spatial conflict task.** Error bars indicate ±1 standard error of the mean (SEM).
