# Supplementary material for: Concurrent measurement of working memory and inhibitory control and their correlations with autistic and ADHD traits in the general population
Source: PLoS One. 2026 Jan 5;21(1):e0339846. doi: 10.1371/journal.pone.0339846 (PMC12768290; doi:10.1371/journal.pone.0339846)
Supplement: S8 Appendix — (DOCX) [file pone.0339846.s008.docx]

**S8 Appendix: Investigating memory and congruency effects based on task order**

A series of Bayesian repeated measures ANOVAs were conducted to explore variations in the memory and congruency effects based on the task order. These analyses were carried out separately for reaction time and accuracy. As mentioned before, in contrast to the Study 1 where all participants started the battery with the flanker task, in this study, 50 participants initiated the task with the flanker task, while the other 50 began with the spatial conflict task. This arrangement was intended to deepen our comprehension of how components of executive functions are influenced by task order. Based on the results from a series of Bayesian repeated measures ANOVAs, although some differences emerged, the effects were not consistent across tasks or measures (reaction time vs. accuracy), suggesting that task order did not systematically affect the results. The results of these analyses are detailed in Tables S8.1 and S8.2.

**Table S8.1. Bayesian repeated measures ANOVA results for the flanker task (both reaction time and accuracy) based on task order.**

|  | RT |
| --- | --- |
| flanker First | 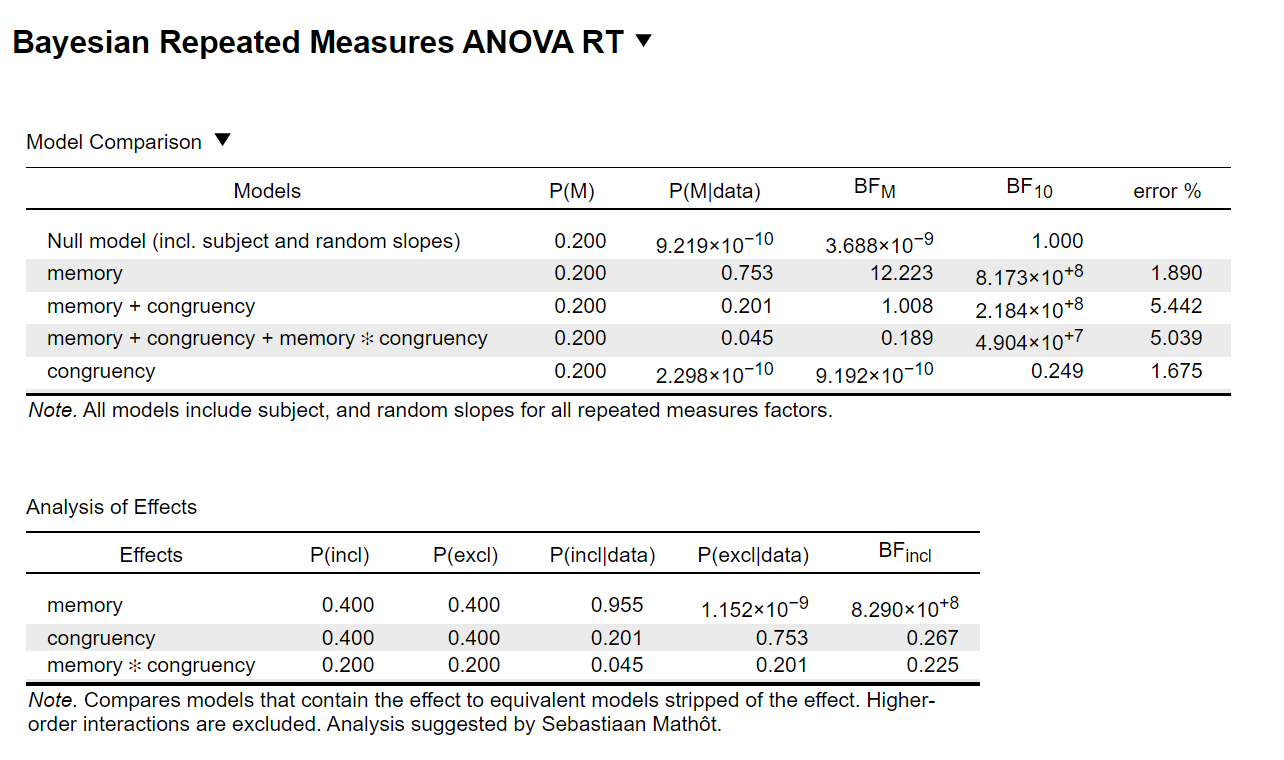 |
| Spatial First | 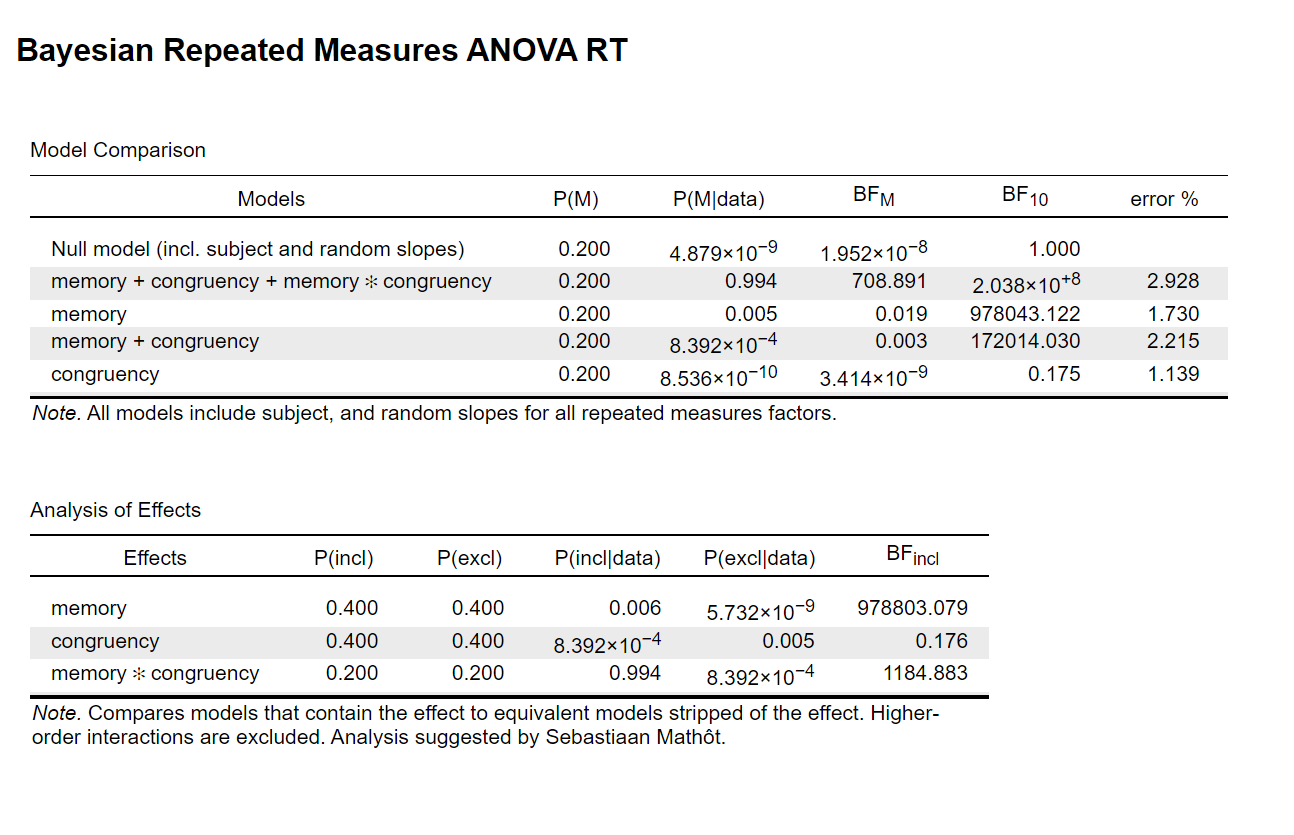 |

|  | Accuracy |
| --- | --- |
| flanker First | 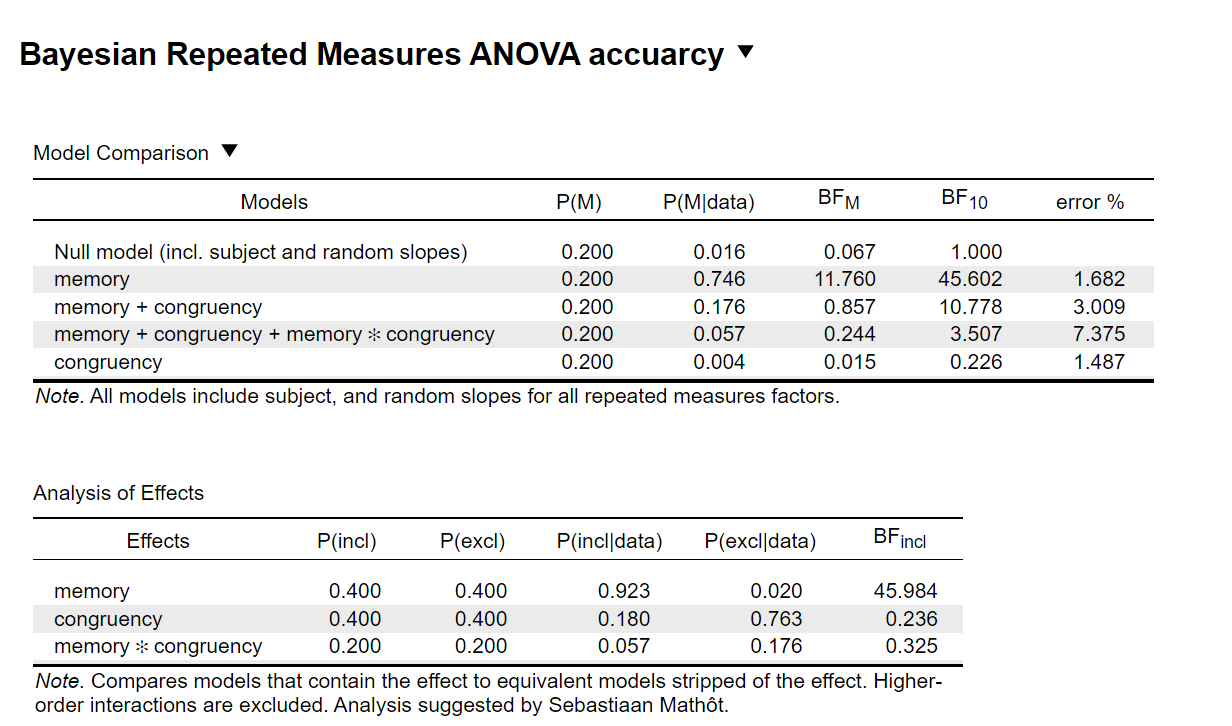 |
| Spatial First | 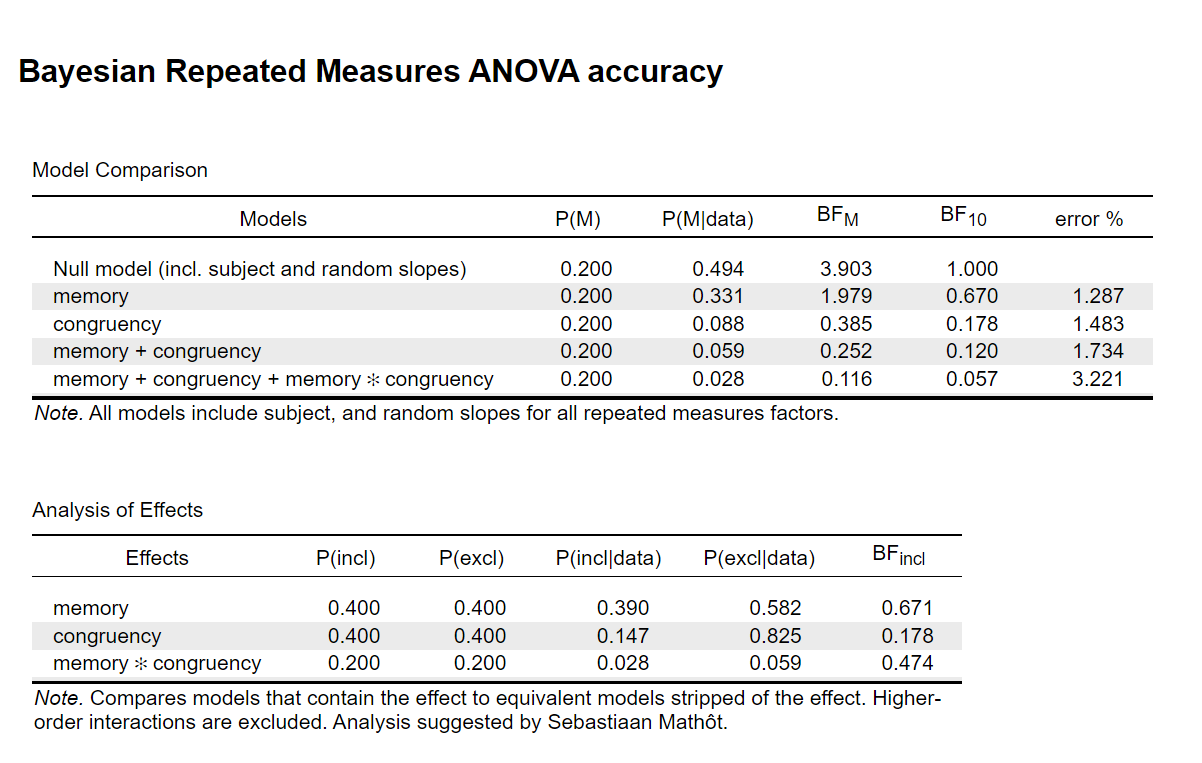 |

**Table S8.2. Bayesian repeated measures ANOVA results for the spatial conflict task (both reaction time and accuracy) based on task order.**

|  | RT |
| --- | --- |
| Spatial First | 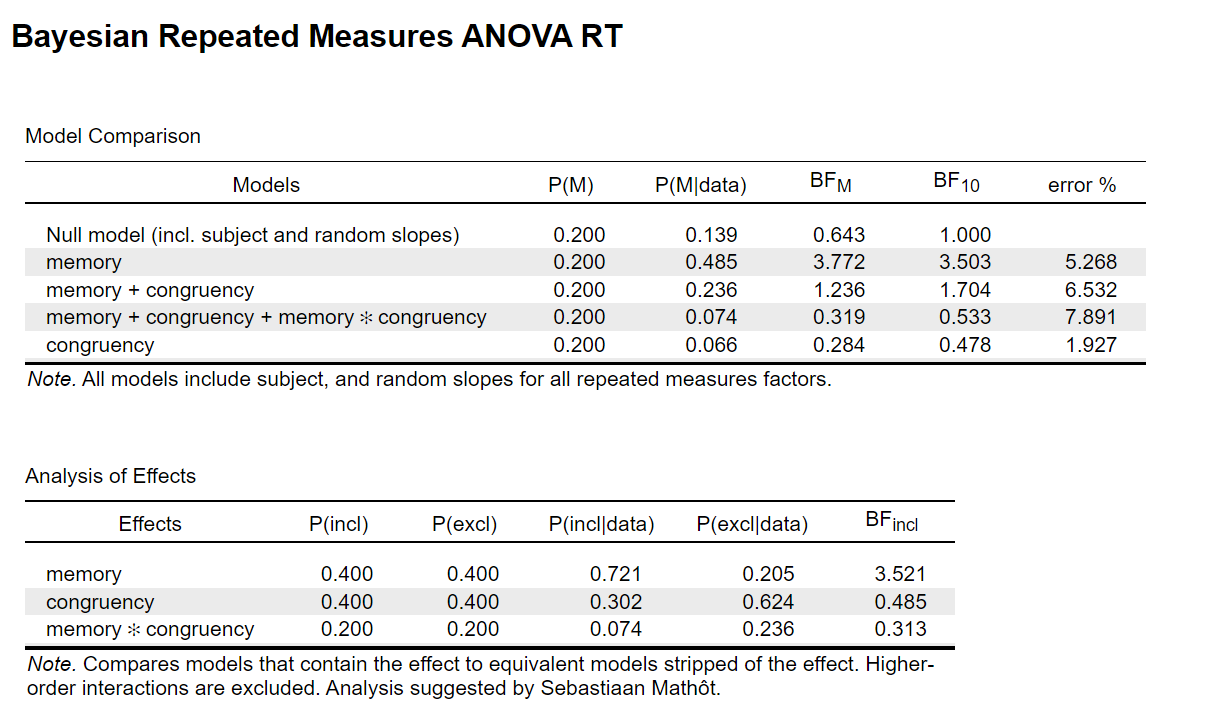 |
| flanker First | 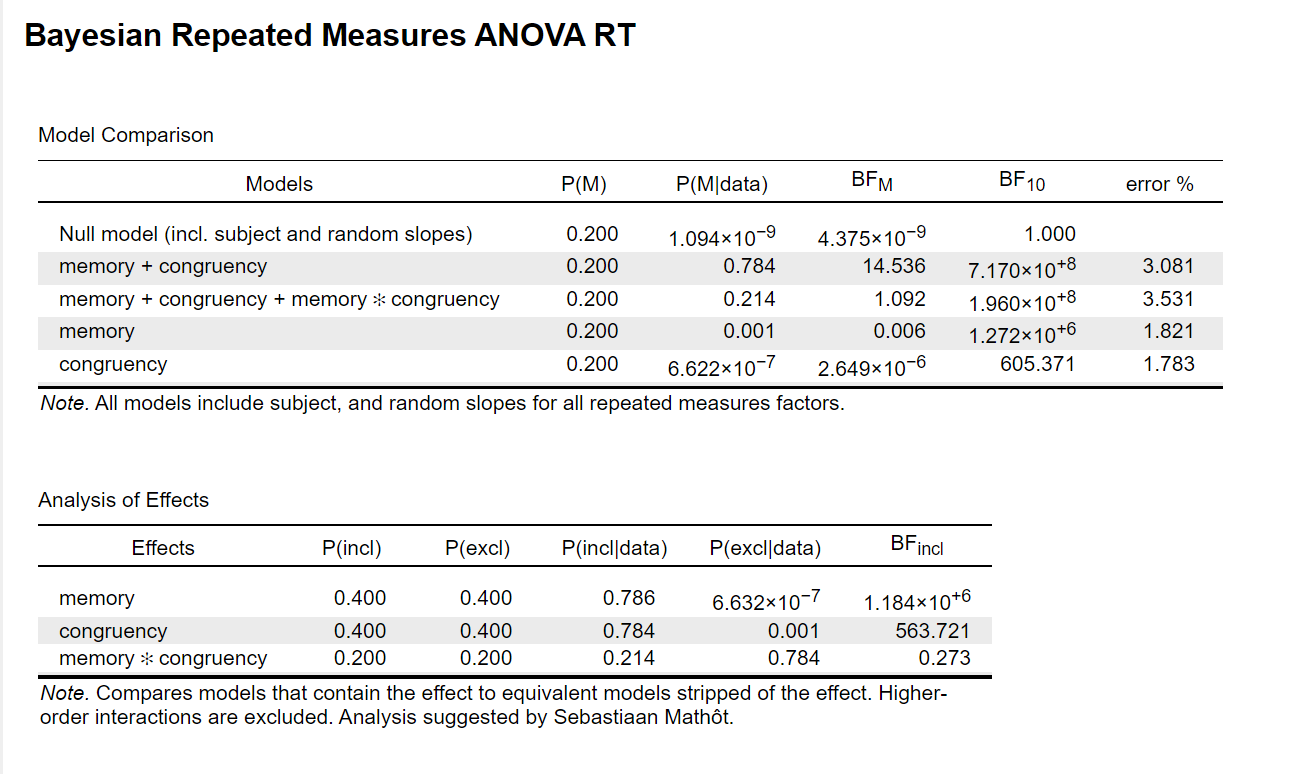 |

|  | Accuracy |
| --- | --- |
| Spatial First | 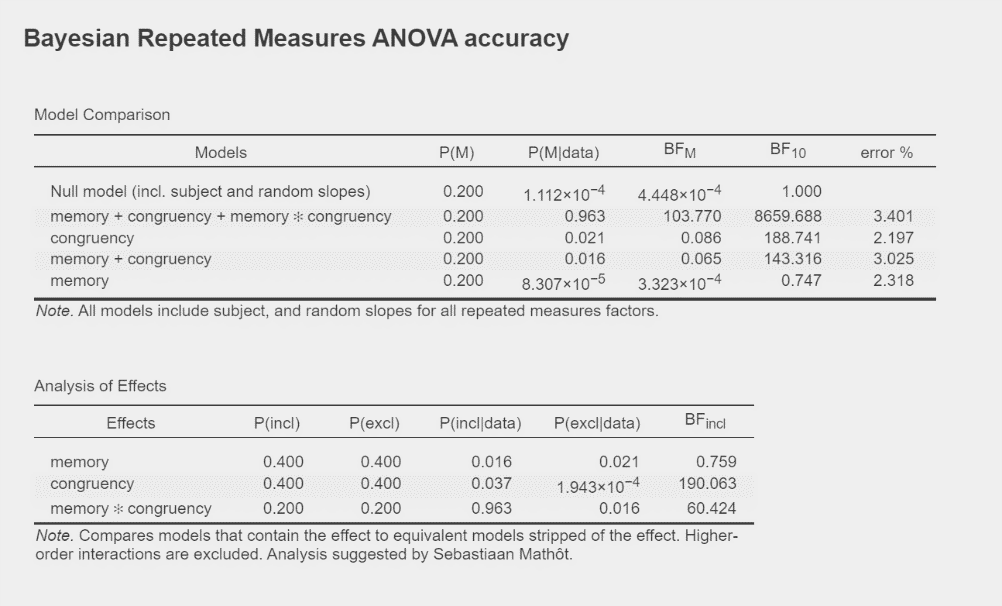 |
| flanker First | 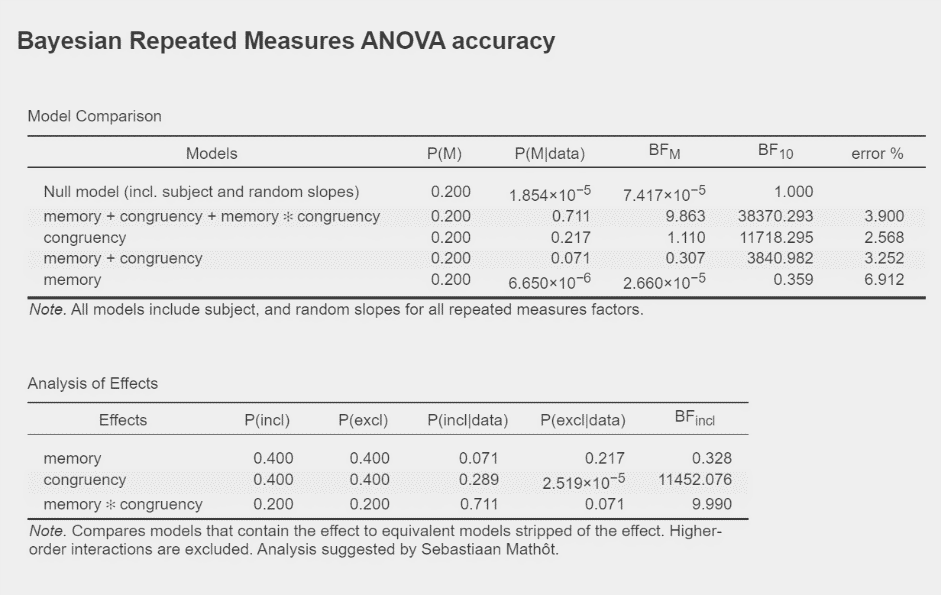 |
