# Supplementary material for: Concurrent measurement of working memory and inhibitory control and their correlations with autistic and ADHD traits in the general population
Source: PLoS One. 2026 Jan 5;21(1):e0339846. doi: 10.1371/journal.pone.0339846 (PMC12768290; doi:10.1371/journal.pone.0339846)
Supplement: S9 Appendix — (DOCX) [file pone.0339846.s009.docx]

**S9 Appendix:** **Correlations between the measures from the cognitive tasks and ASC traits (Study 2)**

**S9a) Descriptive Statistics of ASC traits in study 2**

Descriptive statistics of ASC traits, measured by the Comprehensive Autistic Trait Inventory (CATI), are presented in Table S9.1. The CATI includes six subscales: Social Interactions, Communication, Social Camouflage, Repetitive Behaviours, Cognitive Rigidity, and Sensory Sensitivity. Table 1 presents the descriptive statistics for the total CATI score and each subscale.

**Table S9.1. Descriptive statistics of ASC traits (N=97).**

| CATI subscale | CATI total score | Cognitive rigidity | Communi  cation | Repetitive behaviours | Sensory sensitivity | Social camouflage | Social interactions |
| --- | --- | --- | --- | --- | --- | --- | --- |
| Mean | 121.3 | 22.05 | 15.77 | 21.21 | 18.32 | 21.49 | 22.49 |
| Std. Deviation | 30.54 | 6.234 | 5.409 | 6.748 | 6.959 | 6.733 | 6.835 |
| Minimum | 51.00 | 8.000 | 7.000 | 7.000 | 7.000 | 7.000 | 7.000 |
| Maximum | 191.0 | 35.00 | 35.00 | 35.00 | 33.00 | 35.00 | 35.00 |
|  | | | | | | | |

**S9b) Bayesian analysis results for the correlations between the measures from the cognitive tasks and ASC traits in Study 2**

The results of the Bayesian Pearson correlations between each task performance measure and CATI scores are presented below. Results are shown first for the flanker task (reaction time, accuracy, and inverse efficiency, respectively) and then for the spatial conflict task (reaction time, accuracy, and inverse efficiency, respectively).

**Flanker task**

**S9b1) Bayesian analysis results for the correlations between reaction time in the flanker task and ASC traits.**

Table S9.2 presents the Bayesian correlations between log-transformed reaction times in the flanker task and CATI total and subscale scores, including the posterior mean correlations, 95% credible intervals, and Bayes Factors.

**Table S9.2. Bayesian correlations between log-transformed reaction times of flanker task and CATI total and subscale scores.**

| Condition | CATI subscale | Pearson's r | BF₀₁ | Lower 95% CI | Upper 95% CI |
| --- | --- | --- | --- | --- | --- |
| Low&congruent | CATI total score | -0.141 | 3.090 | -0.327 | 0.059 |
| Low&congruent | Cognitive rigidity | -0.201 | 1.166 | -0.380 | -0.0007 |
| Low&congruent | Communication | -0.256 | 0.338 | -0.428 | -0.058 |
| Low&congruent | Repetitive behaviours | -0.056 | 6.814 | -0.249 | 0.143 |
| Low&congruent | Sensory sensitivity | 0.005 | 7.871 | -0.192 | 0.201 |
| Low&congruent | Social camouflage | -0.048 | 7.089 | -0.241 | 0.151 |
| Low&congruent | Social interactions | -0.148 | 2.822 | -0.333 | 0.053 |
| Low&incongruent | CATI total score | -0.109 | 4.512 | -0.298 | 0.091 |
| Low&incongruent | Cognitive rigidity | -0.147 | 2.862 | -0.332 | 0.054 |
| Low&incongruent | Communication | -0.199 | 1.210 | -0.378 | 0.001 |
| Low&incongruent | Repetitive behaviours | -0.039 | 7.353 | -0.233 | 0.160 |
| Low&incongruent | Sensory sensitivity | -0.004 | 7.872 | -0.200 | 0.193 |
| Low&incongruent | Social camouflage | -0.009 | 7.852 | -0.205 | 0.188 |
| Low&incongruent | Social interactions | -0.146 | 2.897 | -0.331 | 0.055 |
| High&congruent | CATI total score | -0.056 | 6.796 | -0.249 | 0.143 |
| High&congruent | Cognitive rigidity | -0.076 | 6.013 | -0.268 | 0.124 |
| High&congruent | Communication | -0.195 | 1.286 | -0.375 | 0.005 |
| High&congruent | Repetitive behaviours | 0.100 | 4.930 | -0.100 | 0.290 |
| High&congruent | Sensory sensitivity | -0.040 | 7.304 | -0.234 | 0.158 |
| High&congruent | Social camouflage | -0.005 | 7.868 | -0.202 | 0.191 |
| High&congruent | Social interactions | -0.080 | 5.846 | -0.271 | 0.120 |
| High&incongruent | CATI total score | -0.077 | 5.960 | -0.269 | 0.122 |
| High&incongruent | Cognitive rigidity | -0.103 | 4.794 | -0.292 | 0.097 |
| High&incongruent | Communication | -0.191 | 1.407 | -0.371 | 0.009 |
| High&incongruent | Repetitive behaviours | 0.085 | 5.610 | -0.115 | 0.276 |
| High&incongruent | Sensory sensitivity | -0.067 | 6.383 | -0.259 | 0.132 |
| High&incongruent | Social camouflage | -0.025 | 7.644 | -0.221 | 0.172 |
| High&incongruent | Social interactions | -0.091 | 5.336 | -0.281 | 0.109 |

Note. Bayes Factors are BF_01_ values showing the evidence *against* an association

Furthermore, Fig S9.1 shows the Bayesian Pearson correlation matrix between mean reaction time in each flanker condition and CATI total score. The vertical line represents the CATI diagnostic cut-off score of 134, included following reviewer feedback to help contextualize autistic trait levels relative to the diagnostic range. Although this study focuses on autistic traits within the general population and adopts a transdiagnostic perspective, the line might provide a useful reference point for understanding the distribution of scores.

**Fig S9.1. Bayesian Pearson correlation matrix between reaction time (measured in milliseconds) in the flanker task and CATI total score (vertical line indicates CATI diagnostic cut-off score of 134).**

**
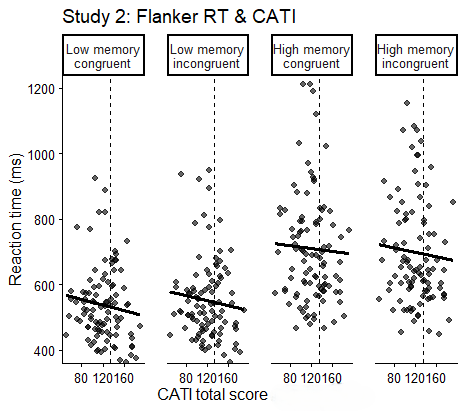
**

**S9b2) Bayesian analysis results for the correlations between accuracy in the flanker task and ASC traits.**

The results of the Bayesian Pearson correlations between accuracy proportions in the flanker task and CATI total and subscale scores are presented in Table S9.3, including the posterior mean correlations, 95% credible intervals, and Bayes Factors.

**Table S9.3. Bayesian correlations between accuracy proportions of flanker task and CATI total and subscale scores.**

| Task Condition | CATI Subscale | Pearson's r | BF_01_ | Lower 95% CI | Upper 95% CI |
| --- | --- | --- | --- | --- | --- |
| Low & Congruent | Total Score | 0.086 | 5.593 | -0.114 | 0.276 |
| Low & Congruent | Cognitive Rigidity | 0.094 | 5.230 | -0.106 | 0.284 |
| Low & Congruent | Communication | 0.008 | 7.853 | -0.189 | 0.204 |
| Low & Congruent | Repetitive Behaviors | 0.110 | 4.470 | -0.090 | 0.299 |
| Low & Congruent | Sensory Sensitivity | -0.023 | 7.690 | -0.218 | 0.175 |
| Low & Congruent | Social Camouflage | 0.074 | 6.085 | -0.125 | 0.266 |
| Low & Congruent | Social Interactions | 0.132 | 3.479 | -0.069 | 0.318 |
| Low & Incongruent | Total Score | -0.007 | 7.862 | -0.203 | 0.190 |
| Low & Incongruent | Cognitive Rigidity | 0.023 | 7.679 | -0.174 | 0.219 |
| Low & Incongruent | Communication | -0.051 | 6.978 | -0.244 | 0.148 |
| Low & Incongruent | Repetitive Behaviors | -0.0006 | 7.878 | -0.197 | 0.196 |
| Low & Incongruent | Sensory Sensitivity | -0.091 | 5.363 | -0.281 | 0.109 |
| Low & Incongruent | Social Camouflage | 0.008 | 7.852 | -0.189 | 0.205 |
| Low & Incongruent | Social Interactions | 0.074 | 6.122 | -0.126 | 0.265 |
| High & Congruent | Total Score | -0.001 | 7.878 | -0.198 | 0.195 |
| High & Congruent | Cognitive Rigidity | 0.061 | 6.617 | -0.138 | 0.254 |
| High & Congruent | Communication | -0.091 | 5.344 | -0.281 | 0.109 |
| High & Congruent | Repetitive Behaviors | 0.026 | 7.641 | -0.172 | 0.221 |
| High & Congruent | Sensory Sensitivity | -0.037 | 7.399 | -0.231 | 0.162 |
| High & Congruent | Social Camouflage | -0.002 | 7.877 | -0.198 | 0.195 |
| High & Congruent | Social Interactions | 0.025 | 7.647 | -0.172 | 0.220 |
| High & Incongruent | Total Score | 0.030 | 7.557 | -0.168 | 0.225 |
| High & Incongruent | Cognitive Rigidity | 0.067 | 6.405 | -0.133 | 0.259 |
| High & Incongruent | Communication | -0.130 | 3.558 | -0.317 | 0.070 |
| High & Incongruent | Repetitive Behaviors | 0.075 | 6.073 | -0.125 | 0.266 |
| High & Incongruent | Sensory Sensitivity | -0.003 | 7.875 | -0.199 | 0.194 |
| High & Incongruent | Social Camouflage | 0.040 | 7.320 | -0.159 | 0.234 |
| High & Incongruent | Social Interactions | 0.066 | 6.430 | -0.133 | 0.258 |

Note. Bayes Factors are BF_01_ values showing the evidence *against* an association

Additionally, Fig S9.2 presents the Bayesian Pearson correlation matrix between accuracy proportions in each flanker condition and CATI total score.

**Fig S9.2. Bayesian Pearson correlation matrix between accuracy proportions in each flanker condition and CATI total score (vertical line indicates CATI diagnostic cut-off score of 134).**

**
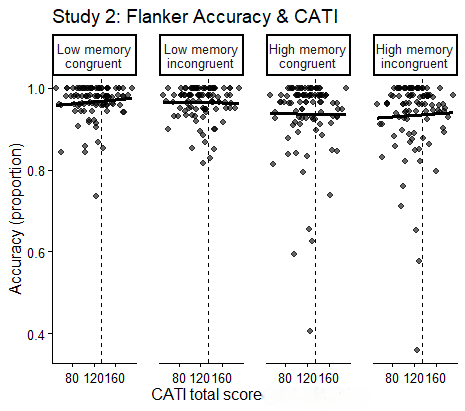
**

**S9b3) Bayesian analysis results for the correlations between inverse efficiency in the flanker task and ASC traits.**

Table S9.4 represents the Bayesian correlations between inverse efficiency scores (IES) in the flanker task and CATI total and subscale scores, reporting the posterior mean correlations, 95% credible intervals, and Bayes Factors.

**Table S9.4. Bayesian correlations between inverse efficiency scores of the flanker task and CATI total and subscale scores**

| Task Condition | CATI subscale | Pearson’s r | BF₀₁ | Lower 95% CI | Upper 95% CI |
| --- | --- | --- | --- | --- | --- |
| Low & Congruent | Total Score | -0.151 | 2.685 | -0.336 | 0.049 |
| Low & Congruent | Cognitive Rigidity | -0.211 | 0.955 | -0.388 | -0.011 |
| Low & Congruent | Communication | -0.240 | 0.502 | -0.414 | -0.041 |
| Low & Congruent | Repetitive Behaviours | -0.076 | 5.996 | -0.268 | 0.123 |
| Low & Congruent | Sensory Sensitivity | 0.010 | 7.841 | -0.187 | 0.206 |
| Low & Congruent | Social Camouflage | -0.062 | 6.600 | -0.254 | 0.138 |
| Low & Congruent | Social Interactions | -0.168 | 2.082 | -0.351 | 0.032 |
| Low & Incongruent | Total Score | -0.101 | 4.877 | -0.291 | 0.099 |
| Low & Incongruent | Cognitive Rigidity | -0.143 | 2.993 | -0.329 | 0.057 |
| Low & Incongruent | Communication | -0.176 | 1.810 | -0.358 | 0.024 |
| Low & Incongruent | Repetitive Behaviours | -0.036 | 7.422 | -0.230 | 0.162 |
| Low & Incongruent | Sensory Sensitivity | 0.015 | 7.800 | -0.183 | 0.210 |
| Low & Incongruent | Social Camouflage | -0.010 | 7.845 | -0.206 | 0.188 |
| Low & Incongruent | Social Interactions | -0.152 | 2.652 | -0.336 | 0.048 |
| High & Congruent | Total Score | -0.049 | 7.030 | -0.243 | 0.149 |
| High & Congruent | Cognitive Rigidity | -0.105 | 4.709 | -0.294 | 0.095 |
| High & Congruent | Communication | -0.122 | 3.905 | -0.310 | 0.078 |
| High & Congruent | Repetitive Behaviours | 0.077 | 5.987 | -0.123 | 0.268 |
| High & Congruent | Sensory Sensitivity | -0.020 | 7.732 | -0.215 | 0.177 |
| High & Congruent | Social Camouflage | -0.004 | 7.872 | -0.200 | 0.193 |
| High & Congruent | Social Interactions | -0.080 | 5.859 | -0.271 | 0.120 |
| High & Incongruent | Total Score | -0.079 | 5.882 | -0.270 | 0.121 |
| High & Incongruent | Cognitive Rigidity | -0.132 | 3.474 | -0.318 | 0.068 |
| High & Incongruent | Communication | -0.093 | 5.250 | -0.283 | 0.107 |
| High & Incongruent | Repetitive Behaviours | 0.041 | 7.298 | -0.158 | 0.235 |
| High & Incongruent | Sensory Sensitivity | -0.054 | 6.863 | -0.248 | 0.145 |
| High & Incongruent | Social Camouflage | -0.038 | 7.363 | -0.232 | 0.160 |
| High & Incongruent | Social Interactions | -0.106 | 4.639 | -0.295 | 0.094 |

Note. Bayes Factors are BF_01_ values showing the evidence *against* an association

Furthermore, the Bayesian Pearson correlation matrix between inverse efficiency scores in each flanker condition and CATI total score is shown in Fig S9.3.

**Fig S9.3. Bayesian Pearson correlation matrix between inverse efficiency scores in each flanker condition and CATI total score (vertical line indicates CATI diagnostic cut-off score of 134).**

**
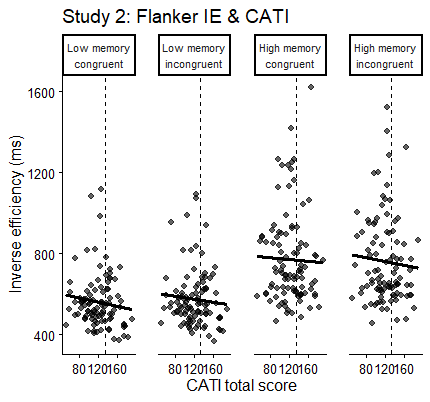
**

**Spatial conflict task**

**S9b4) Bayesian analysis results for the correlations between reaction time in the spatial conflict task and ASC traits.**

Table S9.5 presents the Bayesian Pearson correlations between log-transformed reaction times in the spatial conflict task and CATI total and subscale scores which includes the posterior mean correlations, 95% credible intervals, and Bayes Factors.

**Table S9.5. Bayesian correlations between log-transformed reaction times of the spatial conflict task and CATI total and subscale score.**

| Task condition | CATI subscale | Pearson’s r | BF₁₀ | Lower 95% CI | Upper 95% CI |
| --- | --- | --- | --- | --- | --- |
| Low&congruent | CATI total score | -0.171 | 2.036 | -0.356 | 0.033 |
| Low&congruent | Cognitive rigidity | -0.151 | 2.728 | -0.339 | 0.052 |
| Low&congruent | Communication | -0.324 | 0.053 | -0.488 | -0.127 |
| Low&congruent | Repetitive behaviours | -0.061 | 6.571 | -0.256 | 0.142 |
| Low&congruent | Sensory sensitivity | -0.129 | 3.644 | -0.318 | 0.075 |
| Low&congruent | Social camouflage | -0.058 | 6.676 | -0.253 | 0.145 |
| Low&congruent | Social interactions | -0.128 | 3.691 | -0.317 | 0.076 |
| Low&incongruent | CATI total score | -0.170 | 2.067 | -0.355 | 0.034 |
| Low&incongruent | Cognitive rigidity | -0.176 | 1.868 | -0.361 | 0.027 |
| Low&incongruent | Communication | -0.377 | 0.008 | -0.532 | -0.185 |
| Low&incongruent | Repetitive behaviours | -0.045 | 7.089 | -0.241 | 0.157 |
| Low&incongruent | Sensory sensitivity | -0.087 | 5.509 | -0.280 | 0.116 |
| Low&incongruent | Social camouflage | -0.051 | 6.884 | -0.248 | 0.151 |
| Low&incongruent | Social interactions | -0.124 | 3.853 | -0.314 | 0.079 |
| High&congruent | CATI total score | -0.064 | 6.441 | -0.259 | 0.138 |
| High&congruent | Cognitive rigidity | -0.121 | 3.973 | -0.312 | 0.082 |
| High&congruent | Communication | -0.201 | 1.206 | -0.383 | 0.002 |
| High&congruent | Repetitive behaviours | 0.084 | 5.639 | -0.119 | 0.278 |
| High&congruent | Sensory sensitivity | -0.066 | 6.362 | -0.261 | 0.136 |
| High&congruent | Social camouflage | 0.017 | 7.659 | -0.184 | 0.215 |
| High&congruent | Social interactions | -0.054 | 6.791 | -0.250 | 0.148 |
| High&incongruent | CATI total score | -0.080 | 5.812 | -0.274 | 0.123 |
| High&incongruent | Cognitive rigidity | -0.123 | 3.913 | -0.313 | 0.081 |
| High&incongruent | Communication | -0.236 | 0.591 | -0.413 | -0.034 |
| High&incongruent | Repetitive behaviours | 0.120 | 4.018 | -0.083 | 0.311 |
| High&incongruent | Sensory sensitivity | -0.092 | 5.301 | -0.285 | 0.111 |
| High&incongruent | Social camouflage | -0.017 | 7.655 | -0.216 | 0.183 |
| High&incongruent | Social interactions | -0.073 | 6.092 | -0.268 | 0.130 |

Note. Bayes Factors are BF_01_ values showing the evidence *against* an association

The Bayesian Pearson correlation matrix in Fig S9.4 illustrates the associations between reaction times across the spatial conflict task conditions and the CATI total score.

**Fig S9.4. Bayesian Pearson correlation matrix between reaction times in each spatial conflict condition and CATI total score (vertical line indicates CATI diagnostic cut-off score of 134).**


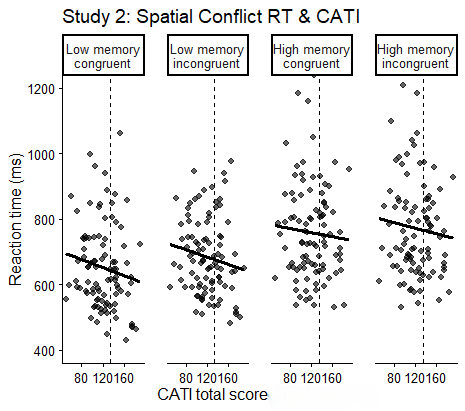


**S9b5) Bayesian analysis results for the correlations between accuracy in the spatial conflict task and ASC traits.**

Table S9.6 summarises the Bayesian Pearson correlations between accuracy proportions in the spatial conflict task and CATI total and subscale scores, including the posterior mean correlations, 95% credible intervals, and Bayes Factors.

**Table S9.6. Bayesian correlations between the spatial conflict task accuracy (proportion correct) and CATI total and subscale scores.**

| Task condition | CATI subscale | Pearson's r | BF₀₁ | Lower 95% CI | Upper 95% CI |
| --- | --- | --- | --- | --- | --- |
| Low&congruent | CATI total score | 0.125 | 3.836 | -0.079 | 0.314 |
| Low&congruent | Cognitive rigidity | 0.096 | 5.106 | -0.107 | 0.289 |
| Low&congruent | Communication | 0.146 | 2.949 | -0.058 | 0.334 |
| Low&congruent | Repetitive behaviours | 0.068 | 6.286 | -0.134 | 0.263 |
| Low&congruent | Sensory sensitivity | 0.144 | 3.030 | -0.060 | 0.332 |
| Low&congruent | Social camouflage | 0.008 | 7.737 | -0.192 | 0.207 |
| Low&congruent | Social interactions | 0.136 | 3.338 | -0.068 | 0.325 |
| Low&incongruent | CATI total score | -0.003 | 7.754 | -0.202 | 0.197 |
| Low&incongruent | Cognitive rigidity | -0.009 | 7.731 | -0.208 | 0.191 |
| Low&incongruent | Communication | 0.063 | 6.495 | -0.140 | 0.258 |
| Low&incongruent | Repetitive behaviours | -0.092 | 5.288 | -0.285 | 0.111 |
| Low&incongruent | Sensory sensitivity | -0.036 | 7.324 | -0.233 | 0.166 |
| Low&incongruent | Social camouflage | -0.028 | 7.495 | -0.225 | 0.173 |
| Low&incongruent | Social interactions | 0.101 | 4.874 | -0.102 | 0.293 |
| High&congruent | CATI total score | -0.154 | 2.619 | -0.341 | 0.049 |
| High&congruent | Cognitive rigidity | -0.117 | 4.186 | -0.307 | 0.087 |
| High&congruent | Communication | -0.137 | 3.321 | -0.325 | 0.067 |
| High&congruent | Repetitive behaviours | -0.132 | 3.501 | -0.322 | 0.071 |
| High&congruent | Sensory sensitivity | -0.116 | 4.233 | -0.306 | 0.088 |
| High&congruent | Social camouflage | -0.165 | 2.252 | -0.350 | 0.039 |
| High&congruent | Social interactions | -0.067 | 6.336 | -0.262 | 0.136 |
| High&incongruent | CATI total score | -0.107 | 4.612 | -0.299 | 0.096 |
| High&incongruent | Cognitive rigidity | -0.040 | 7.211 | -0.237 | 0.161 |
| High&incongruent | Communication | -0.031 | 7.434 | -0.228 | 0.170 |
| High&incongruent | Repetitive behaviours | -0.104 | 4.764 | -0.296 | 0.100 |
| High&incongruent | Sensory sensitivity | -0.122 | 3.955 | -0.312 | 0.082 |
| High&incongruent | Social camouflage | -0.128 | 3.702 | -0.317 | 0.076 |
| High&incongruent | Social interactions | -0.066 | 6.387 | -0.261 | 0.137 |

Note. Bayes Factors are BF_01_ values showing the evidence *against* an association

Furthermore, the Bayesian Pearson correlation matrix in Fig S9.5 depicts the correlations between accuracy proportions across the spatial conflict task conditions and the CATI total score.

**Fig S9.5. Bayesian Pearson correlation matrix between accuracy proportions in each spatial conflict condition and CATI total score (vertical line indicates CATI diagnostic cut-off score of 134).**

**
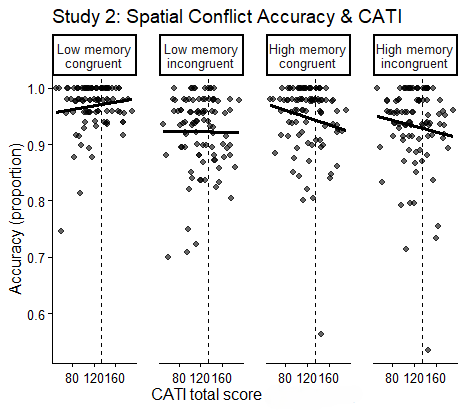
**

**S9b6) Bayesian analysis results for the correlations between inverse efficiency in the spatial conflict task and ASC traits.**

Table S9.7 reports the Bayesian correlations between inverse efficiency scores in the spatial conflict task and CATI total and subscale scores, presenting the posterior mean correlations, 95% credible intervals, and Bayes Factors.

**Table S9.7. Bayesian correlations between log-transformed inverse efficiency scores of spatial conflict task and CATI total and subscale scores.**

| Task condition | CATI subscale | Pearson's r | BF₀₁ | Lower 95% CI | Upper 95% CI |
| --- | --- | --- | --- | --- | --- |
| Low&congruent | CATI total score | -0.189 | 1.505 | -0.372 | 0.014 |
| Low&congruent | Cognitive rigidity | -0.164 | 2.256 | -0.350 | 0.039 |
| Low&congruent | Communication | -0.335 | 0.036 | -0.498 | -0.139 |
| Low&congruent | Repetitive behaviours | -0.073 | 6.081 | -0.268 | 0.129 |
| Low&congruent | Sensory sensitivity | -0.153 | 2.683 | -0.340 | 0.051 |
| Low&congruent | Social camouflage | -0.058 | 6.651 | -0.254 | 0.144 |
| Low&congruent | Social interactions | -0.151 | 2.763 | -0.338 | 0.053 |
| Low&incongruent | CATI total score | -0.153 | 2.675 | -0.340 | 0.051 |
| Low&incongruent | Cognitive rigidity | -0.156 | 2.563 | -0.343 | 0.048 |
| Low&incongruent | Communication | -0.359 | 0.015 | -0.518 | -0.165 |
| Low&incongruent | Repetitive behaviours | -0.005 | 7.748 | -0.204 | 0.195 |
| Low&incongruent | Sensory sensitivity | -0.067 | 6.342 | -0.262 | 0.136 |
| Low&incongruent | Social camouflage | -0.039 | 7.254 | -0.236 | 0.163 |
| Low&incongruent | Social interactions | -0.152 | 2.714 | -0.339 | 0.052 |
| High&congruent | CATI total score | -0.002 | 7.754 | -0.202 | 0.197 |
| High&congruent | Cognitive rigidity | -0.069 | 6.239 | -0.264 | 0.133 |
| High&congruent | Communication | -0.135 | 3.406 | -0.324 | 0.069 |
| High&congruent | Repetitive behaviours | 0.126 | 3.771 | -0.078 | 0.316 |
| High&congruent | Sensory sensitivity | -0.021 | 7.602 | -0.219 | 0.180 |
| High&congruent | Social camouflage | 0.076 | 5.987 | -0.127 | 0.270 |
| High&congruent | Social interactions | -0.023 | 7.574 | -0.221 | 0.178 |
| High&incongruent | CATI total score | -0.025 | 7.547 | -0.223 | 0.176 |
| High&incongruent | Cognitive rigidity | -0.090 | 5.398 | -0.283 | 0.114 |
| High&incongruent | Communication | -0.189 | 1.520 | -0.372 | 0.015 |
| High&incongruent | Repetitive behaviours | 0.146 | 2.921 | -0.057 | 0.334 |
| High&incongruent | Sensory sensitivity | -0.032 | 7.416 | -0.229 | 0.170 |
| High&incongruent | Social camouflage | 0.037 | 7.283 | -0.164 | 0.235 |
| High&incongruent | Social interactions | -0.034 | 7.373 | -0.231 | 0.168 |

Note. Bayes Factors are BF_01_ values showing the evidence *against* an association

The Bayesian Pearson correlation matrix in Fig S9.6 depicts the correlation between inverse efficiency scores across spatial conflict task conditions and the CATI total score.

**Fig S9.6. Bayesian Pearson correlation matrix between inverse efficiency scores in each spatial conflict condition and CATI total score (vertical line indicates CATI diagnostic cut-off score of 134).**

**
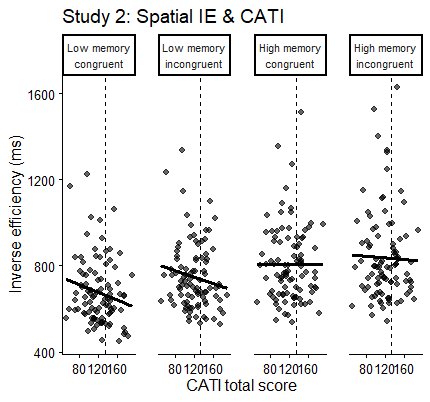
**
