# Supplementary material for: Concurrent measurement of working memory and inhibitory control and their correlations with autistic and ADHD traits in the general population
Source: PLoS One. 2026 Jan 5;21(1):e0339846. doi: 10.1371/journal.pone.0339846 (PMC12768290; doi:10.1371/journal.pone.0339846)
Supplement: S10 Appendix — (DOCX) [file pone.0339846.s010.docx]

**S10 Appendix: Correlations between the measures from the cognitive tasks and ADHD traits (Study 2)**

**S10a) Descriptive Statistics of ADHD traits in study 2**

Descriptive statistics of ADHD traits, measured by the Adult ADHD Self-Report Scale (ASRS), are presented in Table S10.1. The ASRS includes two subscales: inattention and hyperactivity/impulsivity. Table 1 presents the descriptive statistics for the total ASRS score and each subscale.

**Table S10.1. Descriptive statistics of ADHD traits (N=92).**

|  | Total score | Hyperactivity score | Inattentive score |
| --- | --- | --- | --- |
| Mean | 36.47 | 16.29 | 20.17 |
| Std. Deviation | 14.07 | 7.864 | 7.238 |
| Minimum | 2.000 | 0.000 | 1.000 |
| Maximum | 63.00 | 33.00 | 36.00 |
|  | | | |

**S10b) Bayesian analysis results for the correlations between the measures from the cognitive tasks and ADHD traits in Study 2**

The results of the Bayesian Pearson correlations between each task performance measure and ASRS scores are presented below. Results are shown first for the flanker task (reaction time, accuracy, and inverse efficiency, respectively) and then for the spatial conflict task (reaction time, accuracy, and inverse efficiency, respectively).

**Flanker task**

**S10b1) Bayesian analysis results for the correlations between reaction time in the flanker task and ADHD traits.**

Table S10.2 presents the Bayesian correlations between log-transformed reaction times in the flanker task and ASRS total and subscale scores, including the posterior mean correlations, 95% credible intervals, and Bayes Factors.

**Table S10.2. Bayesian correlations between log-transformed reaction times of flanker task and ASRS total and subscale scores.**

| Task condition | ASRS subscale | Pearson's r | BF₀₁ | Lower 95% CI | Upper 95% CI |
| --- | --- | --- | --- | --- | --- |
| Low&congruent | ASRS total score | 0.068 | 6.308 | -0.133 | 0.262 |
| Low&congruent | Hyperactivity score | 0.079 | 5.862 | -0.123 | 0.272 |
| Low&congruent | Inattentive score | 0.047 | 7.059 | -0.154 | 0.242 |
| Low&incongruent | ASRS total score | 0.088 | 5.484 | -0.114 | 0.280 |
| Low&incongruent | Hyperactivity score | 0.097 | 5.051 | -0.105 | 0.289 |
| Low&incongruent | Inattentive score | 0.065 | 6.437 | -0.137 | 0.259 |
| High&congruent | ASRS total score | 0.008 | 7.773 | -0.191 | 0.206 |
| High&congruent | Hyperactivity score | 0.052 | 6.895 | -0.149 | 0.247 |
| High&congruent | Inattentive score | -0.040 | 7.256 | -0.236 | 0.161 |
| High&incongruent | ASRS total score | 0.020 | 7.653 | -0.179 | 0.218 |
| High&incongruent | Hyperactivity score | 0.062 | 6.531 | -0.139 | 0.257 |
| High&incongruent | Inattentive score | -0.028 | 7.529 | -0.225 | 0.172 |

Note. Bayes Factors are BF_01_ values showing the evidence *against* an association.

Furthermore, Fig S10.1 shows the Bayesian Pearson correlation matrix between mean reaction time in each flanker condition and the ASRS total score. Although this study focuses on ADHD traits within the general population and adopts a transdiagnostic perspective, the vertical line marks an ASRS cut-off score of 40, included following reviewer feedback to help contextualize trait levels relative to the diagnostic range. It is important to note that different scoring methods and cut-off values exist for the ASRS in the literature. Some approaches use the two-part structure of the questionnaire (Part A and Part B), with Part A commonly serving as the primary diagnostic screener and Part B as a complementary section (e.g., [1]). Other references report total-score cut-offs, sometimes stratified by gender [2], or provide a general total-score threshold (e.g., [3]). In the present study, we used the cut-off of 40 based on Kessler et al. [3].

**Fig S10.1. Bayesian Pearson correlation matrix between reaction time (measured in milliseconds) in the flanker task and ASRS total score (vertical line indicates ASRS diagnostic cut-off score of 40).**

**
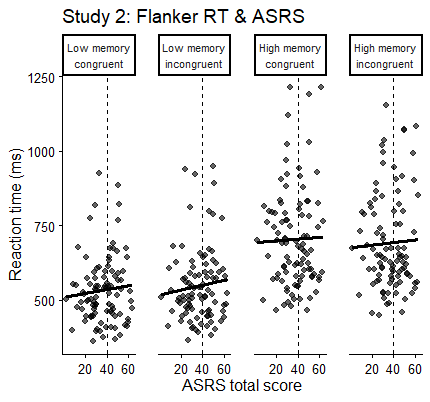
**

**S10b2) Bayesian analysis results for the correlations between accuracy in the flanker task and ADHD traits.**

The results of the Bayesian Pearson correlations between accuracy proportions in the flanker task and ASRS total and subscale scores are presented in Table S10.3, including the posterior mean correlations, 95% credible intervals, and Bayes Factors.

**Table S10.3. Bayesian correlations between accuracy proportions of flanker task and ASRS total and subscale scores.**

| Task condition | ASRS subscale | Pearson's r | BF₀₁ | Lower 95% CI | Upper 95% CI |
| --- | --- | --- | --- | --- | --- |
| Low&congruent | ASRS total score | -0.079 | 5.873 | -0.272 | 0.123 |
| Low&congruent | Hyperactivity score | -0.085 | 5.621 | -0.277 | 0.117 |
| Low&congruent | Inattentive score | -0.061 | 6.573 | -0.256 | 0.140 |
| Low&incongruent | ASRS total score | -0.184 | 1.632 | -0.366 | 0.019 |
| Low&incongruent | Hyperactivity score | -0.157 | 2.488 | -0.343 | 0.045 |
| Low&incongruent | Inattentive score | -0.186 | 1.581 | -0.368 | 0.017 |
| High&congruent | ASRS total score | -0.077 | 5.928 | -0.271 | 0.124 |
| High&congruent | Hyperactivity score | -0.052 | 6.904 | -0.247 | 0.149 |
| High&congruent | inattentive score | -0.094 | 5.208 | -0.286 | 0.108 |
| High&incongruent | ASRS total score | -0.093 | 5.250 | -0.285 | 0.109 |
| High&incongruent | heperactivity_score | -0.066 | 6.375 | -0.261 | 0.135 |
| High&incongruent | Inattentive score | -0.108 | 4.566 | -0.299 | 0.094 |

Note. Bayes Factors are BF_01_ values showing the evidence *against* an association

Additionally, Fig S10.2 presents the Bayesian Pearson correlation matrix between accuracy proportions in each flanker condition and ASRS total score.

**Fig S10.2. Bayesian Pearson correlation matrix between accuracy proportions in each flanker condition and ASRS total score (vertical line indicates ASRS diagnostic cut-off score of 40).**

**
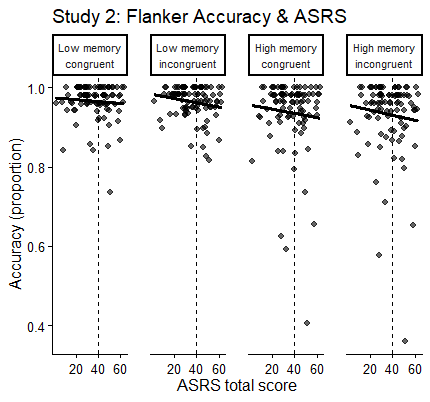
**

**S10b3) Bayesian analysis results for the correlations between inverse efficiency in the flanker task and ADHD traits.**

Table S10.4 represents the Bayesian correlations between inverse efficiency scores (IES) in the flanker task and ASRS total and subscale scores, reporting the posterior mean correlations, 95% credible intervals, and Bayes Factors.

**Table S10.4. Bayesian correlations between inverse efficiency scores of the flanker task and ASRS total and subscale scores.**

| Task condition | ASRS subscale | Pearson's r | BF₀₁ | Lower 95% CI | Upper 95% CI |
| --- | --- | --- | --- | --- | --- |
| Low&congruent | ASRS total score | 0.082 | 5.742 | -0.120 | 0.275 |
| Low&congruent | Hyperactivity score | 0.093 | 5.267 | -0.109 | 0.285 |
| Low&congruent | Inattentive score | 0.059 | 6.668 | -0.143 | 0.253 |
| Low&incongruent | ASRS total score | 0.120 | 4.029 | -0.083 | 0.309 |
| Low&incongruent | Hyperactivity score | 0.124 | 3.863 | -0.079 | 0.313 |
| Low&incongruent | Inattentive score | 0.099 | 4.989 | -0.103 | 0.290 |
| High&congruent | ASRS total score | 0.051 | 6.940 | -0.150 | 0.246 |
| High&congruent | Hyperactivity score | 0.073 | 6.124 | -0.129 | 0.266 |
| High&congruent | Inattentive score | 0.020 | 7.662 | -0.180 | 0.217 |
| High&incongruent | ASRS total score | 0.070 | 6.243 | -0.132 | 0.264 |
| High&incongruent | Hyperactivity score | 0.090 | 5.398 | -0.112 | 0.282 |
| High&incongruent | Inattentive score | 0.038 | 7.288 | -0.162 | 0.235 |

Note. Bayes Factors are BF_01_ values showing the evidence *against* an association

Furthermore, the Bayesian Pearson correlation matrix between inverse efficiency scores in each flanker condition and ASRS total score is shown in Fig S10.3.

**Fig S10.3. Bayesian Pearson correlation matrix between inverse efficiency scores in each flanker condition and ASRS total score (vertical line indicates ASRS diagnostic cut-off score of 40).**

**
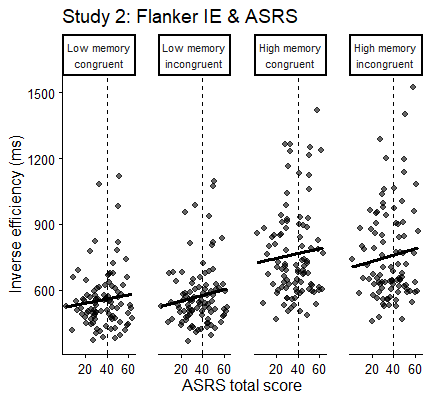
**

**Spatial conflict task**

**S10b4) Bayesian analysis results for the correlations between reaction time in the spatial conflict task and ADHD traits.**

Table S10.5 presents the Bayesian Pearson correlations between log-transformed reaction times in the spatial conflict task and ASRS total and subscale scores which includes the posterior mean correlations, 95% credible intervals, and Bayes Factors.

**Table S10.5. Bayesian correlations between log-transformed reaction times of the spatial conflict task and ASRS total and subscale score.**

| Task condition | ASRS subscale | Pearson's r | BF₀₁ | Lower 95% CI | Upper 95% CI |
| --- | --- | --- | --- | --- | --- |
| Low&congruent | ASRS total score | 0.033 | 7.319 | -0.171 | 0.232 |
| Low&congruent | Hyperactivity score | 0.087 | 5.489 | -0.118 | 0.282 |
| Low&congruent | Inattentive score | -0.031 | 7.358 | -0.231 | 0.172 |
| Low&incongruent | ASRS total score | -0.002 | 7.673 | -0.203 | 0.200 |
| Low&incongruent | Hyperactivity score | 0.052 | 6.796 | -0.152 | 0.251 |
| Low&incongruent | Inattentive score | -0.061 | 6.526 | -0.258 | 0.144 |
| High&congruent | ASRS total score | 0.012 | 7.625 | -0.190 | 0.213 |
| High&congruent | Hyperactivity score | 0.045 | 7.024 | -0.159 | 0.244 |
| High&congruent | Inattentive score | -0.025 | 7.460 | -0.225 | 0.178 |
| High&incongruent | ASRS total score | 0.059 | 6.592 | -0.146 | 0.256 |
| High&incongruent | Hyperactivity score | 0.081 | 5.750 | -0.124 | 0.277 |
| High&incongruent | Inattentive score | 0.026 | 7.444 | -0.177 | 0.226 |

Note. Bayes Factors are BF_01_ values showing the evidence *against* an association

The Bayesian Pearson correlation matrix in Fig S10.4 illustrates the associations between reaction times across the spatial conflict task conditions and the ASRS total score.

**Fig S10.4. Bayesian Pearson correlation matrix between reaction times in each spatial conflict condition and ASRS total score (vertical line indicates ASRS diagnostic cut-off score of 40).**


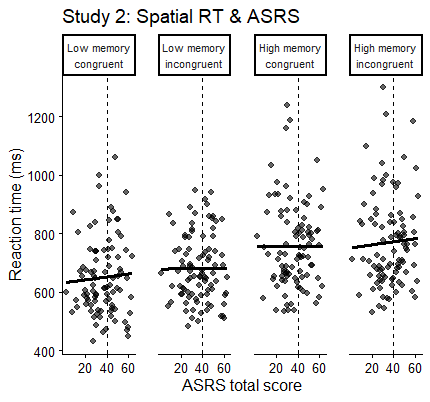


**S10b5) Bayesian analysis results for the correlations between accuracy in the spatial conflict task and ADHD traits.**

Table S10.6 summarises the Bayesian Pearson correlations between accuracy proportions in the spatial conflict task and ASRS total and subscale scores, including the posterior mean correlations, 95% credible intervals, and Bayes Factors.

**Table S10.6. Bayesian correlations between the spatial conflict task accuracy (proportion correct) and ASRS total and subscale scores.**

| Task condition | ASRS subscale | Pearson's r | BF₀₁ | Lower 95% CI | Upper 95% CI |
| --- | --- | --- | --- | --- | --- |
| Low&congruent | ASRS total score | 0.069 | 6.205 | -0.135 | 0.266 |
| Low&congruent | Hyperactivity score | 0.093 | 5.225 | -0.112 | 0.288 |
| Low&congruent | Inattentive score | 0.034 | 7.303 | -0.170 | 0.233 |
| Low&incongruent | ASRS total score | -0.078 | 5.860 | -0.274 | 0.127 |
| Low&incongruent | Hyperactivity score | -0.072 | 6.098 | -0.269 | 0.133 |
| Low&incongruent | Inattentive score | -0.073 | 6.047 | -0.270 | 0.131 |
| High&congruent | ASRS total score | -0.083 | 5.677 | -0.278 | 0.123 |
| High&congruent | Hyperactivity score | -0.075 | 5.971 | -0.272 | 0.130 |
| High&congruent | Inattentive score | -0.079 | 5.841 | -0.275 | 0.126 |
| High&incongruent | ASRS total score | -0.080 | 5.788 | -0.276 | 0.125 |
| High&incongruent | Hyperactivity score | -0.074 | 6.008 | -0.271 | 0.130 |
| High&incongruent | Inattentive score | -0.074 | 6.011 | -0.271 | 0.131 |

Note. Bayes Factors are BF_01_ values showing the evidence *against* an association

Furthermore, the Bayesian Pearson correlation matrix in Fig S10.5 depicts the correlations between accuracy proportions across the spatial conflict task conditions and the ASRS total score.

**Fig S10.5. Bayesian Pearson correlation matrix between accuracy proportions in each spatial conflict condition and ASRS total score (vertical line indicates ASRS diagnostic cut-off score of 40).**

**
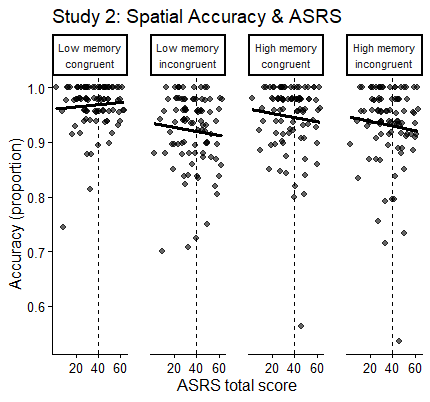
**

**S10b6) Bayesian analysis results for the correlations between inverse efficiency in the spatial conflict task and ADHD traits.**

Table S10.7 reports the Bayesian correlations between inverse efficiency scores in the spatial conflict task and ASRS total and subscale scores, presenting the posterior mean correlations, 95% credible intervals, and Bayes Factors.

**Table S10.7. Bayesian correlations between log-transformed inverse efficiency scores of spatial conflict task and ASRS total and subscale scores.**

| Task condition | ASRS subscale | Pearson's r | BF₀₁ | Lower 95% CI | Upper 95% CI |
| --- | --- | --- | --- | --- | --- |
| Low&congruent | ASRS total score | 0.013 | 7.620 | -0.190 | 0.214 |
| Low&congruent | Hyperactivity score | 0.058 | 6.603 | -0.146 | 0.256 |
| Low&congruent | Inattentive score | -0.039 | 7.181 | -0.238 | 0.165 |
| Low&incongruent | ASRS total score | 0.026 | 7.449 | -0.177 | 0.226 |
| Low&incongruent | Hyperactivity score | 0.071 | 6.160 | -0.134 | 0.267 |
| Low&incongruent | Inattentive score | -0.026 | 7.447 | -0.226 | 0.177 |
| High&congruent | ASRS total score | 0.044 | 7.058 | -0.160 | 0.242 |
| High&congruent | Hyperactivity score | 0.071 | 6.154 | -0.134 | 0.267 |
| High&congruent | Inattentive score | 0.008 | 7.653 | -0.194 | 0.209 |
| High&incongruent | ASRS total score | 0.083 | 5.647 | -0.122 | 0.279 |
| High&incongruent | Hyperactivity score | 0.100 | 4.909 | -0.105 | 0.295 |
| High&incongruent | Inattentive score | 0.053 | 6.788 | -0.151 | 0.251 |

Note. Bayes Factors are BF_01_ values showing the evidence *against* an association

The Bayesian Pearson correlation matrix in Fig S10.6 depicts the correlation between inverse efficiency scores across spatial conflict task conditions and the ASRS total score.

**Fig S10.6. Bayesian Pearson correlation matrix between inverse efficiency scores in each spatial conflict condition and ASRS total score (vertical line indicates ASRS diagnostic cut-off score of 40).**

**
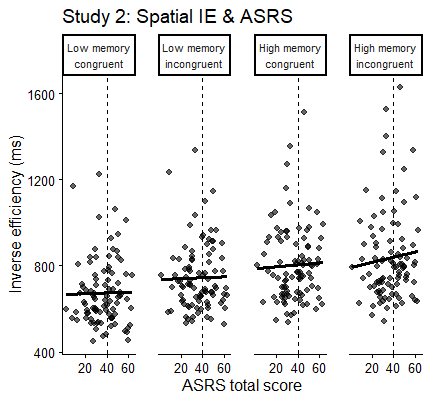
**

**References**

1. Adult A. Self-Report Scale–V1. 1 (ASRS-v1. 1) Symptoms Checklist. World Health Organ. 2003;

2. Olofsdotter S, Fernández-Quintana Á, Sonnby K, Vadlin S. Clinical utility of new cut-off scores for the world health organization ADHD self-report scale among adolescents in psychiatric outpatient care. Int J Clin Health Psychol [Internet]. 2023;23(4):100391. Available from: https://www.sciencedirect.com/science/article/pii/S1697260023000273

3. Kessler RC, Adler L, Ames M, Demler O, Faraone S, Hiripi EVA, et al. The World Health Organization Adult ADHD Self-Report Scale (ASRS): a short screening scale for use in the general population. Psychol Med. 2005;35(2):245–56.
