# Supplementary material for: Concurrent measurement of working memory and inhibitory control and their correlations with autistic and ADHD traits in the general population
Source: PLoS One. 2026 Jan 5;21(1):e0339846. doi: 10.1371/journal.pone.0339846 (PMC12768290; doi:10.1371/journal.pone.0339846)
Supplement: S11 Appendix — (DOCX) [file pone.0339846.s011.docx]

**S11 Appendix: . Partial correlations between cognitive task measures and ASC traits (Study 2)**

Although the preregistration focused on associations between the task measures and the CATI total score, this supplementary appendix provides additional analyses for the six CATI subscales in response to reviewer feedback. These revealed no evidence of a meaningful correlation between the size of any congruency or memory effects and ASC traits on any CATI subscale.

**S11a) Partial correlations between the flanker task measures and ASC traits**

Table S11.1 presents the results of the Bayesian regression analyses (equivalent to partial correlations) examining associations between the two CATI subscales (Social Interactions, Communication, Social Camouflage, Repetitive Behaviours, Cognitive Rigidity, and Sensory Sensitivity) and performance on the flanker task. It includes results for correlations between incongruent-trial performance and each CATI subscale (controlling for congruent trials) and between high-memory performance and each CATI subscale (controlling for low-memory trials), reported separately for RT, accuracy, and inverse efficiency.

**Table S11.1. Partial correlations between CATI subscales and the flanker task performance.**

| CATI subscale | Partial correlation design | RT | Accuracy | Inverse efficiency |
| --- | --- | --- | --- | --- |
| Social Interactions | Incongruent-trial performance (controlling for congruent trials) | BF₍incl₎ = 0.004 Mean = 6.019×10⁻⁷ 95% CI = [0.000, 0.000] | BF₍incl₎ = 0.009 Mean = 2.149×10⁻⁶ 95% CI = [0.000, 0.000] | BF₍incl₎ = 0.006 Mean = −1.620×10⁻⁶ 95% CI = [0.000, 0.000] |
|  | High-memory performance (controlling for low-memory trials) | BF₍incl₎ = 0.043 Mean = 1.090×10⁻⁴ 95% CI = [0.000, 0.000] | BF(incl)=0.109 mean=9.282×10⁻⁵ 95%CI=[-0.001,0.001] | BF₍incl₎ = 0.053 Mean = 9.478×10⁻⁵ 95% CI = [0.000, 0.000] |
| Communication | Incongruent-trial performance (controlling for congruent trials) | BF₍incl₎ = 0.010 Mean = 1.342×10⁻⁵ 95% CI = [0.000, 0.000] | BF₍incl₎ = 0.010 Mean = −3.880×10⁻⁶ 95% CI = [0.000, 0.000] | BF₍incl₎ = 0.010 Mean = 1.466×10⁻⁵ 95% CI = [0.000, 0.000] |
|  | High-memory performance (controlling for low-memory trials) | BF₍incl₎ = 0.043 Mean = −1.163×10⁻⁴ 95% CI = [0.000, 0.000] | BF(incl)=0.245 mean=  -5.696×10⁻⁴ 95%CI=[-0.005,0] | BF₍incl₎ = 0.069 Mean = −2.819×10⁻⁴ 95% CI = [0.000, 0.000] |
| Social Camouflage | Incongruent-trial performance (controlling for congruent trials) | BF₍incl₎ = 0.006 Mean = 3.548×10⁻⁶ 95% CI = [0.000, 0.000] | BF₍incl₎ = 0.010 Mean = 3.211×10⁻⁶ 95% CI = [0.000, 0.000] | BF₍incl₎ = 0.006 Mean = 1.473×10⁻⁶ 95% CI = [0.000, 0.000] |
|  | High-memory performance (controlling for low-memory trials) | BF₍incl₎ = 0.042 Mean = −4.526×10⁻⁵ 95% CI = [0.000, 0.000] | BF(incl)=0.107 mean=-1.914×10⁻⁵ 95%CI=[-0.002,0.000] | BF₍incl₎ = 0.056 Mean = −4.922×10⁻⁵ 95% CI = [0.000, 0.000] |
| Repetitive Behaviours | Incongruent-trial performance (controlling for congruent trials) | BF₍incl₎ = 0.004 Mean = −9.888×10⁻⁸ 95% CI = [0.000, 0.000] | BF₍incl₎ = 0.009 Mean = 2.060×10⁻⁶ 95% CI = [0.000, 0.000] | BF₍incl₎ = 0.006 Mean = −1.644×10⁻⁶ 95% CI = [0.000, 0.000] |
|  | High-memory performance (controlling for low-memory trials) | BF₍incl₎ = 0.202 Mean = 0.001 95% CI = [0.000, 0.009] | BF(incl)=0.110 mean=9.467×10⁻⁵ 95%CI=[-0.0007861,0.001] | BF₍incl₎ = 0.252 Mean = 0.002 95% CI = [0.000, 0.010] |
| Cognitive Rigidity | Incongruent-trial performance (controlling for congruent trials) | BF₍incl₎ = 0.004 Mean = 2.472×10⁻⁸ 95% CI = [0.000, 0.000] | BF₍incl₎ = 0.008 Mean = 5.370×10⁻⁷ 95% CI = [0.000, 0.000] | BF₍incl₎ = 0.006 Mean = −1.857×10⁻⁶ 95% CI = [0.000, 0.000] |
|  | High-memory performance (controlling for low-memory trials) | BF₍incl₎ = 0.051 Mean = 1.683×10⁻⁴ 95% CI = [0.000, 0.001] | BF(incl)=0.113 mean=1.191×10⁻⁴ 95%CI=[-0.00074,0.002] | BF₍incl₎ = 0.059 Mean = 1.263×10⁻⁴ 95% CI = [0.000, 0.000] |
| Sensory Sensitivity | Incongruent-trial performance (controlling for congruent trials) | BF₍incl₎ = 0.004 Mean = −2.179×10⁻⁷ 95% CI = [0.000, 0.000] | BF₍incl₎ = 0.009 Mean = 2.156×10⁻⁶ 95% CI = [0.000, 0.000] | BF₍incl₎ = 0.006 Mean = −2.077×10⁻⁶ 95% CI = [0.000, 0.000] |
|  | High-memory performance (controlling for low-memory trials) | BF₍incl₎ = 0.060 Mean = −2.735×10⁻⁴ 95% CI = [0.000, 0.001] | BF(incl)=0.110 mean=-2.395×10⁻⁶ 95%CI=[-0.001,0.001] | BF₍incl₎ = 0.071 Mean = −2.468×10⁻⁴ 95% CI = [0.000, 0.000] |

Note, BF₍inclusion₎ is the Bayes factor comparing models that include a predictor against models that exclude it.

**S11b) Partial correlations between the spatial conflict task measures and ASC traits**

Table S11.2 summarises the Bayesian regression analyses (serving as partial correlations) examining how the two CATI subscales relate to performance on the spatial conflict task. It presents the associations between the CATI subscales and both incongruent-trial performance (adjusted for congruent trials) and high-memory performance (adjusted for low-memory trials), shown separately for RT, accuracy, and inverse efficiency.

**Table S11.2. Partial correlations between CATI subscales and the spatial conflict task performance.**

| CATI subscale | Partial correlation design | RT | Accuracy | Inverse Efficiency |
| --- | --- | --- | --- | --- |
| Social Interactions | Incongruent-trial performance (controlling for congruent trials, age, gender) | BF₍incl₎=0.008 Mean=−6.319×10⁻⁷ CI=[0.000,0.000] | BF₍incl₎=0.024 Mean=2.510×10⁻⁶ CI=[0.000,0.000] | BF₍incl₎=0.007 Mean=−6.806×10⁻⁷ CI=[0.000,0.000] |
|  | High-memory performance (controlling for low-memory trials, age, gender) | BF₍incl₎=0.044 Mean=1.951×10⁻⁵ CI=[0.000,0.000] | BF₍incl₎=0.109 Mean=−8.432×10⁻⁵ CI=[−0.001,1.098×10⁻⁴] | BF₍incl₎=0.058 Mean=6.986×10⁻⁵ CI=[−2.047×10⁻⁴,0.001] |
| Communication | Incongruent-trial performance (controlling for congruent trials, age, gender) | BF₍incl₎=0.052 Mean=−1.349×10⁻⁴ CI=[−5.576×10⁻⁴,0.000] | BF₍incl₎=0.035 Mean=3.163×10⁻⁵ CI=[0.000,0.000] | BF₍incl₎=0.028 Mean=−6.278×10⁻⁵ CI=[0.000,0.000] |
|  | High-memory performance (controlling for low-memory trials, age, gender) | BF₍incl₎=0.046 Mean=−2.690×10⁻⁵ CI=[0.000,0.000] | BF₍incl₎=0.112 Mean=−1.201×10⁻⁴ CI=[−0.002,0.000] | BF₍incl₎=0.056 Mean=4.114×10⁻⁵ CI=[0.000,0.000] |
| Social Camouflage | Incongruent-trial performance (controlling for congruent trials, age, gender) | BF₍incl₎=0.010 Mean=−6.239×10⁻⁶ CI=[0.000,0.000] | BF₍incl₎=0.025 Mean=4.675×10⁻⁶ CI=[0.000,0.000] | BF₍incl₎=0.008 Mean=−3.302×10⁻⁶ CI=[0.000,0.000] |
|  | High-memory performance (controlling for low-memory trials, age, gender) | BF₍incl₎=0.047 Mean=3.441×10⁻⁶ CI=[0.000,7.272×10⁻⁵] | BF₍incl₎=0.118 Mean=−1.019×10⁻⁴ CI=[−0.002,0.000] | BF₍incl₎=0.063 Mean=8.534×10⁻⁵ CI=[−1.134×10⁻⁴,0.002] |
| Repetitive Behaviours | Incongruent-trial performance (controlling for congruent trials, age, gender) | BF₍incl₎=0.041 Mean=8.139×10⁵ CI=[0.000,0.000] | BF₍incl₎=0.036 Mean=−2.617×10⁻⁵ CI=[0.000,0.000] | BF₍incl₎=0.026 Mean=4.352×10⁻⁵ CI=[0.000,0.000] |
|  | High-memory performance (controlling for low-memory trials, age, gender) | BF₍incl₎=0.198 Mean=8.885×10⁻⁴ CI=[0.000,0.007] | BF₍incl₎=0.084 Mean=−2.406×10⁻⁵ CI=[−6.626×10⁻⁴,1.313×10⁵] | BF₍incl₎=0.251 Mean=0.001 CI=[0.000,0.008] |
| Cognitive Rigidity | Incongruent-trial performance (controlling for congruent trials, age, gender) | BF₍incl₎=0.009 Mean=−1.672×10⁻⁶ CI=[0.000,0.000] | BF₍incl₎=0.025 Mean=5.134×10⁻⁶ CI=[0.000,0.000] | BF₍incl₎=0.007 Mean=−9.092×10⁻⁷ CI=[0.000,0.000] |
|  | High-memory performance (controlling for low-memory trials, age, gender) | BF₍incl₎=0.065 Mean=−1.994×10⁻⁴ CI=[−0.001,2.206×10⁻⁴] | BF₍incl₎=0.081 Mean=−1.466×10⁻⁵ CI=[−9.078×10⁻⁴,5.060×10⁻⁵] | BF₍incl₎=0.069 Mean=−1.938×10⁻⁴ CI=[−0.003,5.666×10⁻⁵] |
| Sensory Sensitivity | Incongruent-trial performance (controlling for congruent trials, age, gender) | BF₍incl₎=0.010 Mean=5.746×10⁻⁶ CI=[0.000,0.000] | BF₍incl₎=0.049 Mean=−4.684×10⁻⁵ CI=[0.000,0.000] | BF₍incl₎=0.008 Mean=3.823×10⁻⁶ CI=[0.000,0.000] |
|  | High-memory performance (controlling for low-memory trials, age, gender) | BF₍incl₎=0.053 Mean=−9.112×10⁻⁵ CI=[0.000,0.000] | BF₍incl₎=0.116 Mean=−9.711×10⁻⁵ CI=[−0.001,3.984×10⁻⁵] | BF₍incl₎=0.057 Mean=−4.584×10⁻⁵ CI=[0.000,7.239×10⁵] |

Note, BF₍inclusion₎ is the Bayes factor comparing models that include a predictor against models that exclude it.
