# Supplementary material for: Concurrent measurement of working memory and inhibitory control and their correlations with autistic and ADHD traits in the general population
Source: PLoS One. 2026 Jan 5;21(1):e0339846. doi: 10.1371/journal.pone.0339846 (PMC12768290; doi:10.1371/journal.pone.0339846)
Supplement: S12 Appendix — (DOCX) [file pone.0339846.s012.docx]

**S12 Appendix: Partial correlations between cognitive task measures and ASC traits with age and gender as covariates (Study 2)**

Although these analyses were not preregistered, this supplementary appendix includes additional Bayesian regression analyses (equivalent to partial correlations) examining the associations between ASC traits and task performance with age and gender included as covariates. These analyses showed no meaningful correlations between the size of any congruency or memory effects and ASC traits across the CATI subscales after adjusting for these covariates (see Tables S12.1 and S12.2 below).

**S12a) Partial correlations between the flanker task measures and ASC traits (with age & gender as covariates)**

Table S12.1 presents the results of the Bayesian regression analyses (equivalent to partial correlations) examining associations between CATI scores and performance on the flanker task. The table reports correlations between incongruent-trial performance and CATI scores (controlling for congruent trials), and between high-memory performance and CATI scores (controlling for low-memory trials), with age and gender included as covariates. Results are shown separately for RT, accuracy, and inverse efficiency.

**Table S12.1. Partial correlations between CATI and the flanker task performance (with age & gender as covariates).**

| CATI subscale | Partial correlation design | RT | Accuracy | Inverse efficiency |
| --- | --- | --- | --- | --- |
| Total score | Incongruent-trial performance (controlling for congruent trials, age, gender) | BF₍incl₎=0.007 Mean=2.699×10⁻⁷ 95%CI=[0.000,0.000] | BF₍incl₎=0.012 Mean=4.718×10⁻⁷ 95%CI=[0.000,0.000] | BF₍incl₎=0.009 Mean=−2.318×10⁻⁷ 95%CI=[0.000,0.000] |
|  | High-memory performance (controlling for low-memory trials, age, gender) | BF₍incl₎=0.107 Mean=3.456×10⁻⁵ 95%CI=[−0.001,0.001] | BF(incl)=0.169 mean=−3.639×10⁻⁵ 95%CI=[−6.421×10⁻⁴,7.575×10⁻⁴] | BF₍incl₎=0.132 Mean=−1.335×10⁻⁵ 95%CI=[−9.756×10⁻⁴,0.002] |
| Social Interactions | Incongruent-trial performance (controlling for congruent trials, age, gender) | BF₍incl₎=0.006 Mean=5.048×10⁻⁷ 95%CI=[0.000,0.000] | BF₍incl₎=0.012 Mean=2.068×10⁻⁶ 95%CI=[0.000,0.000] | BF₍incl₎=0.009 Mean=−2.071×10⁻⁶ 95%CI=[0.000,0.000] |
|  | High-memory performance (controlling for low-memory trials, age, gender) | BF₍incl₎=0.077 Mean=1.689×10⁻⁴ 95%CI=[−4.696×10⁻⁴,0.003] | BF(incl)=0.160 mean=1.238×10⁻⁴ 95%CI=[−7.123×10⁻⁴,0.002] | BF₍incl₎=0.094 Mean=1.296×10⁻⁴ 95%CI=[−4.628×10⁻⁴,0.004] |
| Communication | Incongruent-trial performance (controlling for congruent trials, age, gender) | BF₍incl₎=0.013 Mean=1.606×10⁻⁵ 95%CI=[0.000,0.000] | BF₍incl₎=0.013 Mean=−3.928×10⁻⁶ 95%CI=[0.000,0.000] | BF₍incl₎=0.014 Mean=1.757×10⁻⁵ 95%CI=[0.000,0.000] |
|  | High-memory performance (controlling for low-memory trials, age, gender) | BF₍incl₎=0.076 Mean=−1.536×10⁻⁴ 95%CI=[−0.003,0.002] | BF(incl)=0.282 mean=−5.584×10⁻⁴ 95%CI=[−0.005,1.028×10⁻⁴] | BF₍incl₎=0.114 Mean=−3.441×10⁻⁴ 95%CI=[−0.005,0.003] |
| Social Camouflage | Incongruent-trial performance (controlling for congruent trials, age, gender) | BF₍incl₎=0.008 Mean=4.372×10⁻⁶ 95%CI=[0.000,0.000] | BF₍incl₎=0.014 Mean=3.820×10⁻⁶ 95%CI=[0.000,0.000] | BF₍incl₎=0.009 Mean=2.300×10⁻⁶ 95%CI=[0.000,0.000] |
|  | High-memory performance (controlling for low-memory trials, age, gender) | BF₍incl₎=0.077 Mean=−7.887×10⁻⁵ 95%CI=[−3.429×10⁻⁴,0.003] | BF(incl)=0.158 mean=−2.053×10⁻⁵ 95%CI=[−0.002,0.001] | BF₍incl₎=0.100 Mean=−6.730×10⁻⁵ 95%CI=[−0.004,5.863×10⁻⁴] |
| Repetitive Behaviours | Incongruent-trial performance (controlling for congruent trials, age, gender) | BF₍incl₎=0.006 Mean=−6.935×10⁻⁷ 95%CI=[0.000,0.000] | BF₍incl₎=0.013 Mean=3.093×10⁻⁶ 95%CI=[0.000,0.000] | BF₍incl₎=0.009 Mean=−3.056×10⁻⁶ 95%CI=[0.000,0.000] |
|  | High-memory performance (controlling for low-memory trials, age, gender) | BF₍incl₎=0.447 Mean=0.002 95%CI=[−1.839×10⁻⁴,0.011] | BF(incl)=0.161 mean=1.252×10⁴ 95%CI=[−0.001,0.002] | BF₍incl₎=0.588 Mean=0.003 95%CI=[0.000,0.012] |
| Cognitive Rigidity | Incongruent-trial performance (controlling for congruent trials, age, gender) | BF₍incl₎=0.006 Mean=−9.429×10⁻⁷ 95%CI=[0.000,0.000] | BF₍incl₎=0.011 Mean=3.788×10⁻⁷ 95%CI=[0.000,0.000] | BF₍incl₎=0.009 Mean=−3.200×10⁻⁶ 95%CI=[0.000,0.000] |
|  | High-memory performance (controlling for low-memory trials, age, gender) | BF₍incl₎=0.092 Mean=2.712×10⁻⁴ 95%CI=[0.000,0.005] | BF(incl)=0.166 mean=1.702×10⁻⁴ 95%CI=[−0.001,0.002] | BF₍incl₎=0.106 Mean=1.901×10⁻⁴ 95%CI=[−2.381×10⁻⁴,0.004] |
| Sensory Sensitivity | Incongruent-trial performance (controlling for congruent trials, age, gender) | BF₍incl₎=0.007 Mean=−2.625×10⁻⁶ 95%CI=[0.000,0.000] | BF₍incl₎=0.012 Mean=1.615×10⁻⁶ 95%CI=[0.000,0.000] | BF₍incl₎=0.009 Mean=−3.960×10⁻⁶ 95%CI=[0.000,0.000] |
|  | High-memory performance (controlling for low-memory trials, age, gender) | BF₍incl₎=0.106 Mean=−4.457×10⁻⁴ 95%CI=[−0.005,0.000] | BF(incl)=0.157 mean=4.170×10⁻⁵ 95%CI=[−0.002,0.001] | BF₍incl₎=0.118 Mean=−3.254×10⁻⁴ 95%CI=[−0.006,6.708×10⁻⁴] |

Note, BF₍inclusion₎ is the Bayes factor comparing models that include a predictor against models that exclude it.

**S12b) Partial correlations between the spatial conflict task measures and ASC traits (with age & gender as covariates)**

Table S12.2 summarises the correlations between CATI scores and performance on the spatial conflict task. It includes associations for incongruent-trial performance (controlling for congruent trials) and for high-memory performance (controlling for low-memory trials), with age and gender entered as covariates. Results are presented separately for RT, accuracy, and inverse efficiency.

**Table S12.2. Partial correlations between CATI subscales and spatial conflict task performance (with age & gender as covariates).**

| CATI subscale | Partial correlation design | RT | Accuracy | Inverse efficiency |
| --- | --- | --- | --- | --- |
| Total score | Incongruent-trial performance (controlling for congruent trials, age, gender) | BF₍incl₎=0.020 Mean=−2.324×10⁻⁶ 95%CI=[0.000,0.000] | BF₍incl₎=0.056 Mean=3.913×10⁻⁶ 95%CI=[0.000,0.000] | BF₍incl₎=0.015 Mean=−1.445×10⁻⁶ 95%CI=[0.000,0.000] |
|  | High-memory performance (controlling for low-memory trials, age, gender) | BF₍incl₎=0.106 Mean=−3.711×10⁻⁵ 95%CI=[−0.001,0.001] | BF₍incl₎=0.238 Mean=−6.661×10⁻⁵ 95%CI=[−7.418×10⁻⁴,0.000] | BF₍incl₎=0.133 Mean=4.701×10⁵ 95%CI=[−1.602×10⁻⁴,0.002] |
| Social Interactions | Incongruent-trial performance (controlling for congruent trials, age, gender) | BF₍incl₎=0.014 Mean=−1.10×10⁻⁶ 95%CI=[0.000,0.000] | BF₍incl₎=0.024 Mean=5.955×10⁻⁶ 95%CI=[0.000,0.000] | BF₍incl₎=0.014 Mean=−1.47×10⁻⁶ 95%CI=[0.000,0.000] |
|  | High-memory performance (controlling for low-memory trials, age, gender) | BF₍incl₎=0.081 Mean=3.750×10⁻⁵ 95%CI=[0.000,0.002] | BF₍incl₎=0.169 Mean=−1.012×10⁻⁴ 95%CI=[−0.002,2.476×10⁻⁴] | BF₍incl₎=0.102 Mean=9.903×10⁵ 95%CI=[0.000,0.004] |
| Communication | Incongruent-trial performance (controlling for congruent trials, age, gender) | BF₍incl₎=0.080 Mean=−2.069×10⁻⁴ 95%CI=[−0.002,0.000] | BF₍incl₎=0.062 Mean=5.598×10⁻⁵ 95%CI=[0.000,0.000] | BF₍incl₎=0.044 Mean=−9.878×10⁻⁵ 95%CI=[−1.345×10⁻⁵,0.000] |
|  | High-memory performance (controlling for low-memory trials, age, gender) | BF₍incl₎=0.082 Mean=−5.093×10⁻⁵ 95%CI=[−0.001,0.002] | BF₍incl₎=0.172 Mean=−1.571×10⁻⁴ 95%CI=[−0.003,1.321×10⁻⁴] | BF₍incl₎=0.098 Mean=4.852×10⁵ 95%CI=[−0.001,0.005] |
| Social Camouflage | Incongruent-trial performance (controlling for congruent trials, age, gender) | BF₍incl₎=0.018 Mean=−1.363×10⁵ 95%CI=[0.000,0.000] | BF₍incl₎=0.048 Mean=1.378×10⁵ 95%CI=[0.000,0.000] | BF₍incl₎=0.013 Mean=−7.449×10⁶ 95%CI=[0.000,0.000] |
|  | High-memory performance (controlling for low-memory trials, age, gender) | BF₍incl₎=0.083 Mean=−4.064×10⁶ 95%CI=[−0.002,3.404×10⁵] | BF₍incl₎=0.216 Mean=−1.947×10⁴ 95%CI=[−0.003,0.000] | BF₍incl₎=0.112 Mean=1.463×10⁴ 95%CI=[−1.026×10⁵,0.005] |
| Repetitive Behaviours | Incongruent-trial performance (controlling for congruent trials, age, gender) | BF₍incl₎=0.068 Mean=1.381×10⁴ 95%CI=[−1.593×10⁻⁵,0.002] | BF₍incl₎=0.066 Mean=−4.964×10⁵ 95%CI=[−4.202×10⁻⁴,3.129×10⁻⁵] | BF₍incl₎=0.042 Mean=7.535×10⁵ 95%CI=[0.000,0.000] |
|  | High-memory performance (controlling for low-memory trials, age, gender) | BF₍incl₎=0.320 Mean=0.001 95%CI=[0.000,0.008] | BF₍incl₎=0.139 Mean=−2.713×10⁻⁵ 95%CI=[−0.002,2.581×10⁻⁴] | BF₍incl₎=0.408 Mean=0.002 95%CI=[0.000,0.009] |
| Cognitive Rigidity | Incongruent-trial performance (controlling for congruent trials, age, gender) | BF₍incl₎=0.014 Mean=−3.611×10⁶ 95%CI=[0.000,0.000] | BF₍incl₎=0.047 Mean=1.119×10⁵ 95%CI=[0.000,0.000] | BF₍incl₎=0.012 Mean=−2.351×10⁶ 95%CI=[0.000,0.000] |
|  | High-memory performance (controlling for low-memory trials, age, gender) | BF₍incl₎=0.114 Mean=−3.359×10⁻⁴ 95%CI=[−0.005,0.000] | BF₍incl₎=0.132 Mean=1.458×10⁵ 95%CI=[−0.002,7.460×10⁻⁵] | BF₍incl₎=0.127 Mean=−3.907×10⁻⁴ 95%CI=[−0.005,2.402×10⁻⁴] |
| Sensory Sensitivity | Incongruent-trial performance (controlling for congruent trials, age, gender) | BF₍incl₎=0.015 Mean=5.666×10⁶ 95%CI=[0.000,0.000] | BF₍incl₎=0.124 Mean=−1.504×10⁻⁴ 95%CI=[−0.002,1.455×10⁻⁵] | BF₍incl₎=0.012 Mean=3.033×10⁶ 95%CI=[0.000,0.000] |
|  | High-memory performance (controlling for low-memory trials, age, gender) | BF₍incl₎=0.095 Mean=−1.733×10⁴ 95%CI=[−0.003,0.002] | BF₍incl₎=0.171 Mean=−1.141×10⁴ 95%CI=[−0.002,7.425×10⁶] | BF₍incl₎=0.103 Mean=−1.106×10⁴ 95%CI=[−2.143×10⁵,0.004] |

Note, BF₍inclusion₎ is the Bayes factor comparing models that include a predictor against models that exclude it.
