# Supplementary material for: Concurrent measurement of working memory and inhibitory control and their correlations with autistic and ADHD traits in the general population
Source: PLoS One. 2026 Jan 5;21(1):e0339846. doi: 10.1371/journal.pone.0339846 (PMC12768290; doi:10.1371/journal.pone.0339846)
Supplement: S13 Appendix — (DOCX) [file pone.0339846.s013.docx]

**S13 Appendix: Partial correlations between cognitive task measures and ADHD traits (Study 2)**

Although the preregistration focused on associations between the task measures and the ASRS total score, this supplementary appendix provides additional analyses for the two ASRS subscales in response to reviewer feedback. These revealed no evidence of a meaningful correlation between the size of any congruency or memory effects and ADHD traits on any ASRS subscale.

**S13a) Partial correlations between the flanker task measures and ADHD traits**

Table S13.1 presents the results of the Bayesian regression analyses (equivalent to partial correlations) examining associations between the two ASRS subscales (inattentive and hyperactivity) and performance on the flanker task. It includes results for correlations between incongruent-trial performance and each ASRS subscale (controlling for congruent trials) and between high-memory performance and each ASRS subscale (controlling for low-memory trials), reported separately for RT, accuracy, and inverse efficiency.

**Table S13.1. Partial correlations between ASRS subscales and the flanker task performance.**

| ASRS subscale | Partial correlation design | RT | Accuracy | Inverse efficiency |
| --- | --- | --- | --- | --- |
| Inattentive | Incongruent-trial performance (controlling for congruent trials, age, gender) | BF₍incl₎=0.019 Mean=1.509×10⁻⁵ 95%CI=[0.000,0.000] | BF₍incl₎=0.066 Mean=-4.057×10⁻⁵ 95%CI=[-4.460×10⁻⁴,1.753×10⁻⁵] | BF₍incl₎=0.061 Mean=8.647×10⁻⁵ 95%CI=[0.000,0.001] |
|  | High-memory performance (controlling for low-memory trials, age, gender) | BF₍incl₎=0.180 Mean=-9.899×10⁻⁴ 95%CI=[-0.009,9.558×10⁻⁶] | BF(incl)=0.212 mean=−1.470×10⁻⁴ 95%CI=[−0.003,0.001] | BF₍incl₎=0.207 Mean=-0.001 95%CI=[-0.009,7.768×10⁻⁴] |
| Hyperactivity | Incongruent-trial performance (controlling for congruent trials, age, gender) | BF₍incl₎=0.014 Mean=3.920×10⁻⁶ 95%CI=[0.000,0.000] | BF₍incl₎=0.038 Mean=-4.307×10⁻⁶ 95%CI=[0.000,0.000] | BF₍incl₎=0.032 Mean=1.370×10⁻⁵ 95%CI=[0.000,0.000] |
|  | High-memory performance (controlling for low-memory trials, age, gender) | BF₍incl₎=0.126 Mean=1.907×10⁻⁴ 95%CI=[-0.002,0.000] | BF(incl)=0.204 mean=1.214×10⁻⁵ 95%CI=[−0.001,0.0001829] | BF₍incl₎=0.160 Mean=2.693×10⁻⁴ 95%CI=[0.000,0.003] |

Note, BF₍inclusion₎ is the Bayes factor comparing models that include a predictor against models that exclude it.

**S13b) Partial correlations between the spatial conflict task measures and ADHD traits**

Table S13.2 summarises the Bayesian regression analyses (serving as partial correlations) examining how the two ASRS subscales relate to performance on the spatial conflict task. It presents the associations between the ASRS subscales and both incongruent-trial performance (adjusted for congruent trials) and high-memory performance (adjusted for low-memory trials), shown separately for RT, accuracy, and inverse efficiency.

**Table S13.2. Partial correlations between ASRS subscales and the spatial conflict task performance.**

| ASRS subscale | Partial correlation design | RT | Accuracy | Inverse efficiency |
| --- | --- | --- | --- | --- |
| Inattentive | Incongruent-trial performance (controlling for congruent trials, age, gender) | BF₍incl₎=0.026 Mean=2.645×10⁻⁵ 95%CI=[0.000,0.000] | BF₍incl₎=0.072 Mean=1.935×10⁻⁶ 95%CI=[-3.903×10⁻⁴, 0.000] | BF₍incl₎=0.025 Mean=2.904×10⁻⁵ 95%CI=[0.000,0.000] |
|  | High-memory performance (controlling for low-memory trials, age, gender) | BF₍incl₎=0.109 Mean=2.897×10⁻⁴ 95%CI=[-0.001,0.003] | BF₍incl₎=0.165 Mean=-1.600×10⁻⁴ 95%CI=[-0.002,8.050×10⁻⁵] | BF₍incl₎=0.151 Mean=6.209×10⁻⁴ 95%CI=[-1.512×10⁻⁴,0.007] |
| Hyperactivity | Incongruent-trial performance (controlling for congruent trials, age, gender) | BF₍incl₎=0.021 Mean=-6.205×10⁻⁶ 95%CI=[0.000,0.000] | BF₍incl₎=0.080 Mean=-2.498×10⁻⁵ 95%CI=[0.000, 0.000] | BF₍incl₎=0.021 Mean=-8.037×10⁻⁶ 95%CI=[0.000,0.000] |
|  | High-memory performance (controlling for low-memory trials, age, gender) | BF₍incl₎=0.101 Mean=-8.077×10⁻⁵ 95% CI=[-0.002,3.472×10⁻⁴] | BF₍incl₎=0.155 Mean=4.440×10⁻⁵ 95% CI=[-2.440×10⁻⁵,8.796×10⁻⁴] | BF₍incl₎=0.137 Mean=-1.787×10⁻⁴ 95%CI=[-0.002,0.000] |

Note, BF₍inclusion₎ is the Bayes factor comparing models that include a predictor against models that exclude it.
