# Supplementary material for: Concurrent measurement of working memory and inhibitory control and their correlations with autistic and ADHD traits in the general population
Source: PLoS One. 2026 Jan 5;21(1):e0339846. doi: 10.1371/journal.pone.0339846 (PMC12768290; doi:10.1371/journal.pone.0339846)
Supplement: S14 Appendix — (DOCX) [file pone.0339846.s014.docx]

**S14 Appendix: Partial correlations between cognitive task measures and ADHD traits with age and gender as covariates (Study 2)**

Although these analyses were not preregistered, this supplementary appendix includes additional Bayesian regression analyses (equivalent to partial correlations) examining the associations between ADHD traits and task performance with age and gender included as covariates. These analyses showed no meaningful correlations between the size of any congruency or memory effects and ADHD traits across the ASRS subscales after adjusting for these covariates (see Tables S14.1 and S14.2 below).

**S14a) Partial correlations between the flanker task measures and ADHD traits (with age & gender as covariates)**

Table S14.1 presents the results of the Bayesian regression analyses (equivalent to partial correlations) examining associations between ASRS scores and performance on the flanker task. The table reports correlations between incongruent-trial performance and ASRS scores (controlling for congruent trials), and between high-memory performance and ASRS scores (controlling for low-memory trials), with age and gender included as covariates. Results are shown separately for RT, accuracy, and inverse efficiency.

**Table S14.1. Partial correlations between ASRS and the flanker task performance (with age & gender as covariates).**

| ASRS subscale | Partial correlation design | RT | Accuracy | Inverse efficiency |
| --- | --- | --- | --- | --- |
| Total score | Incongruent-trial performance (controlling for congruent trials, age, gender) | BF₍incl₎=0.023 Mean=5.689×10⁻⁶ 95% CI=[0.000,0.000] | BF₍incl₎=0.074 Mean=-1.079×10⁻⁵ 95% CI=[-1.992×10⁻⁴,0.000] | BF₍incl₎=0.062 Mean=2.932×10⁻⁵ 95% CI=[-2.478×10⁻⁵,4.895×10⁻⁴] |
|  | High-memory performance (controlling for low-memory trials, age, gender) | BF₍incl₎=0.207 Mean=1.336×10⁻⁴ 95% CI=[-0.003,0.005] | BF(incl)=0.294 mean=1.035×10^-5^ 95%CI=[-0.001,0.002] | BF₍incl₎=0.241 Mean=2.344×10⁻⁴ 95% CI=[-0.003,0.007] |
| Inattentive | Incongruent-trial performance (controlling for congruent trials, age, gender) | BF₍incl₎=0.024 Mean=1.778×10⁻⁵ 95%CI=[0.000,0.000] | BF₍incl₎=0.097  Mean=-5.936×10⁻⁵ 95%CI=[-5.563×10⁻⁴,0.000] | BF₍incl₎ = 0.079  Mean = 1.09 × 10⁻⁴  95% CI=[0.000, 0.001] |
|  | High-memory performance (controlling for low-memory trials, age, gender) | BF₍incl₎=0.251  Mean=-0.001  95%CI=[-0.012,7.913×10⁻⁴] | BF(incl)=0.296 mean=-1.232×10^-4^  95%CI=[-0.004,0.002] | BF₍incl₎ = 0.273  Mean = -0.001  95% CI=[-0.013, 7.09 × 10⁻⁴] |
| Hyperactivity | Incongruent-trial performance (controlling for congruent trials, age, gender) | BF₍incl₎=0.020 Mean=5.073×10⁻⁶ 95%CI=[0.000,0.000] | BF₍incl₎=0.056  Mean=-2.673×10⁻⁶ 95%CI=[0.000,0.000] | BF₍incl₎ = 0.046  Mean = 1.56 × 10⁻⁵  95% CI=[0.000, 0.000] |
|  | High-memory performance (controlling for low-memory trials, age, gender) | BF₍incl₎=0.191 Mean=2.862×10⁻⁴ 95%CI=[-0.002,0.002] | BF(incl)=0.293 mean=2.608×10^-5^ 95%CI=[-0.001,0.001] | BF₍incl₎ = 0.273  Mean = -0.001  95% CI=[-0.013, 7.09 × 10⁻⁴] |

Note, BF₍inclusion₎ is the Bayes factor comparing models that include a predictor against models that exclude it.

**S14b) Partial correlations between the spatial conflict task measures and ADHD traits (with age & gender as covariates)**

Table S14.2 summarises the correlations between ASRS scores and performance on the spatial conflict task. It includes associations for incongruent-trial performance (controlling for congruent trials) and for high-memory performance (controlling for low-memory trials), with age and gender entered as covariates. Results are presented separately for RT, accuracy, and inverse efficiency.

**Table S14.2. Partial correlations between ASRS subscales and spatial conflict task performance (with age & gender as covariates).**

| ASRS subscale | Partial correlation design | RT | Accuracy | Inverse efficiency |
| --- | --- | --- | --- | --- |
| Total score | Incongruent-trial performance (controlling for congruent trials, age, gender) | BF₍incl₎=0.034 Mean=-4.253×10⁻⁶ 95% CI=[0.000,0.000] | BF₍incl₎=0.147 Mean=-4.451×10⁻⁵ 95% CI=[-6.874×10⁻⁴,3.279×10⁻⁴] | BF₍incl₎=0.032 Mean=-6.554×10⁻⁶ 95% CI=[0.000,0.000] |
|  | High-memory performance (controlling for low-memory trials, age, gender) | BF₍incl₎=0.165 Mean=-8.755×10⁻⁵ 95% CI=[-0.002,0.002] | BF₍incl₎=0.240 Mean=5.551×10⁻⁵ 95% CI=[-4.711×10⁻⁴,0.002] | BF₍incl₎=0.223 Mean=-2.070×10⁻⁴ 95% CI=[-0.005,0.001] |
| Inattentive | Incongruent-trial performance (controlling for congruent trials, age, gender) | BF₍incl₎=0.038 Mean=3.71×10⁻⁵ 95%CI=[0.000,0.000] | BF₍incl₎ = 0.135  Mean = −6.916×10⁻⁶  95% CI = [−0.001, 6.360×10⁻⁵] | BF₍incl₎=0.036 Mean=3.944×10⁻⁵ 95%CI=[0.000,0.000] |
|  | High-memory performance (controlling for low-memory trials, age, gender) | BF₍incl₎ = 0.173  Mean = 4.359×10⁻⁴  95% CI = [−9.730×10⁻⁴, 0.005] | BF₍incl₎=0.245 Mean=−2.154×10⁻⁴ 95%CI=[−0.002,7.958×10⁻⁴] | BF(incl)=0.237 Mean=9.174×10⁻⁴ 95%CI=[-0.001,0.009] |
| Hyperactivity | Incongruent-trial performance (controlling for congruent trials, age, gender) | BF₍incl₎=0.033 Mean=−9.85×10⁻⁶ 95%CI=[0.000,0.000] | BF₍incl₎ = 0.149 Mean = −4.726×10⁻⁵  95% CI = [−7.472×10⁻⁴, 1.553×10⁻⁴] | BF₍incl₎=0.033 Mean=-1.283×10⁻⁵ 95%CI=[0.000,0.000] |
|  | High-memory performance (controlling for low-memory trials, age, gender) | BF₍incl₎ = 0.165  Mean = −1.334×10⁻⁴  95% CI = [−0.002, 2.904×10⁻⁴] | BF₍incl₎=0.242 Mean=7.467×10⁻⁵ 95%CI=[−5.499×10⁻⁴,0.001] | BF(incl)=0.224 Mean=-2.893×10⁻⁴ 95%CI=[-0.003,0.002] |

Note, BF₍inclusion₎ is the Bayes factor comparing models that include a predictor against models that exclude it.
