# Supplementary material for: Concurrent measurement of working memory and inhibitory control and their correlations with autistic and ADHD traits in the general population
Source: PLoS One. 2026 Jan 5;21(1):e0339846. doi: 10.1371/journal.pone.0339846 (PMC12768290; doi:10.1371/journal.pone.0339846)
Supplement: Attachment — Submitted filename: Response to Reviewers.docx [file pone.0339846.s016.docx]

**Feedback from Reviewer 1**

Reviewer 1’s comments have been interpreted and categorised into four main areas: Introduction, Methodological, Statistical Analysis, and Interpretation of Results. Each of these areas is summarises and addressed below.

- **Introduction**

Reviewer 1 raised several points regarding the Introduction. We have grouped these comments into two main categories, and they have been addressed throughout the revised Introduction.

1. **Clarifying the Neurodiversity Approach and Addressing the Oversimplification and Overgeneralization of Executive Function in Neurodivergent Populations**
a) Oversimplification of Neurodiverse Conditions: The introduction treats neurodevelopmental conditions as homogeneous, failing to account for individual variability, such as the presence of compensatory mechanisms in high-functioning neurodivergent individuals or those with dual exceptionality. This omission leads to an overly reductive portrayal of EF deficits in these populations.
b) Misrepresentation of EF in ASC Populations: The claim that all individuals with ASC exhibit EF deficits ignores evidence of preserved or enhanced EF in certain contexts. It also neglects the role of environmental and compensatory factors. Moreover, the absence of a robust control group limits the validity of these assertions.
c) Inadequate Consideration of Co-occurring Conditions: While discussing ASC-ADHD overlap, the manuscript neglects other relevant conditions, such as anxiety, depression, or sensory processing disorders, that may confound EF measures. Furthermore, equating genetic overlap with functional equivalence oversimplifies the complexity of shared EF deficits.
d) Inconsistent Framing of Autistic Traits: By broadening its scope to include subclinical autistic traits, the manuscript does not clarify whether these traits directly influence EF or reflect normal cognitive variability. Without robust controls, this approach risks conflating variability in the general population with EF deficits specific to ASC.

**This has been addressed by better explaining the adopted neurodiversity perspective in the introduction. The revised text highlights individual differences in EF, acknowledges the presence of compensatory mechanisms, and references evidence of preserved EF in some autistic individuals. Contextual and environmental factors are also considered.**

**Regarding the lack of a control group, we now explain more clearly the benefits of taking a non-categorical approach to examining neurodiversity within the general population instead of making comparisons with a control group. We also note here how this approach aligns well with the sense of the reviewer’s Points A to C above). We trust that this justifies our approach sufficiently, but we have also highlighted (p. 38-39) the benefits of future work including both individuals with and without formal diagnoses and measuring potential confounding factors to statistically control for these.**

**Regarding the consideration of co-occurring conditions (Point C), there is a related danger that a strong focus on as anxiety and depression would push things back towards a more traditional diagnostic framing. While we fully appreciate that this is not the reviewer’s intention, this would again be in contrast to the dimensional, neurodiversity-informed perspective underpinning the current work. The revised text therefore clarifies our position by presenting co-occurring features as part of a broader, integrated neurodiversity framework rather than as separate categorical diagnoses. Our suggestion of controlling for individual variation on continuous measures of anxiety and depression in future work (p. 39) also directly addresses this concern.**

**The revised text also clarifies that subclinical traits are considered along a continuum within the general population, and that EF variability is interpreted within this broader neurodiversity framework, rather than being attributed solely to clinical thresholds (point D).**

2. **Lack of Specificity in EF Subcomponents**
a) The manuscript does not specify which executive function subcomponents are affected across conditions.

**The revised introduction now outlines the EF subcomponents most commonly impacted within the neurodiverse groups that form the focus of this paper.**

- **Methodological Concerns**

a) Task Randomization and Order: Study 1 employed a fixed task order (Flanker first, followed by Spatial Conflict), potentially introducing fatigue or practice effects. Study 2 counterbalanced task order to address this, but the inconsistency undermines direct comparisons between studies.

**The two studies were designed to be complementary rather than directly comparable. However, we did analyse potential task order effects in Study 2 (see supplementary material S6). While some differences emerged, the effects were not consistent across tasks or measures (reaction time vs. accuracy), suggesting that task order did not systematically bias the results.**

b) Sample Representativeness: The sample comprises only neurotypical participants, limiting generalizability to neurodivergent populations. Furthermore, the absence of detailed demographic data (e.g., age, education, cultural background) limits interpretation of how individual variability may have influenced results.

**In the revised Discussion, we emphasise that the sample consisted of participants who were not selected on the basis of any diagnosis and that we therefore examined correlations in the general population between ASD/ADHD traits and performance on our cognitive tasks. While we highlight the benefits of this approach, we do now explicitly note that this will limit the generalisability of the findings to clinically diagnosed neurodivergent populations (p. 34).**

**Regarding demographics, age data were collected for both studies. In Study 1, we also recorded educational background, as participants were undergraduate psychology students from the University of Bristol, and this information is now added to the Participant section of Study 1 (p. 11).**

c) Working Memory Manipulation: The increased stimulus mapping rules in Study 2 ostensibly heightened working memory demands, but without direct verification (e.g., recall testing), it remains unclear whether participants internalized these rules. This undermines the validity of the manipulation.

**While the novel design of the task for concurrently measuring working memory and inhibition did not include an explicit recall test, the structure of the task required participants to memorize the stimulus–response rules in order to respond correctly. Participants completed guided practice trials that provided accuracy feedback and repeated incorrect responses to support memorization before the main trials. Moreover, a pre-registered exclusion criterion (minimum 60% accuracy on low memory/congruent trials) ensured that only participants who had successfully memorized the rules were included in the analysis. The fact that nearly all participants met this threshold, alongside clear and meaningful reaction time differences observed between low and high memory load conditions in both tasks (particularly in Study 2, where the numbers of rules which needed to be memorized was increased), provides strong evidence that participants were indeed processing and applying the rules as intended (Strong evidence for memory effects in reaction times were observed in both tasks across both studies, though in Study 1, the effect in the spatial task was limited to incongruent trials, while in Study 2 it was more consistent).**

**However, we acknowledge that the task has not been formally validated against existing and accepted working memory measures (without reiterating our concerns over the validity of these ‘accepted’ measures), and this has now been stated as a limitation in the revised manuscript (p. 34) that future work can address.**

d) Task Modifications and Validation: The Flanker and Spatial Conflict tasks were modified to include memory components. However, no prior validation studies were presented, raising concerns about whether these tasks adequately measure the intended constructs (e.g., working memory-inhibitory control interaction).

**The tasks used in this study were based on well-established paradigms. The Spatial Conflict task was adapted from the classic Simon task, and the Flanker task was based on the Eriksen Flanker paradigm (both commonly used to assess interference control and response inhibition). Our versions simply incorporated an additional memory load component to explore the interaction between working memory and inhibitory control, following the approach used in Jarrold et al. (2023).**

**Although the tasks were modified, they consistently produced the expected effects: Both memory and congruency effects were observed in the Spatial Conflict task across studies, although the memory effect in Study 1 was limited to incongruent trials. Similarly, in the Flanker task, both effects were present, though in Study 2 the congruency effect emerged only under the low memory condition. The presence of these clear and robust effects, particularly in reaction time, provides strong evidence that the tasks effectively captured the intended constructs, including working memory demands, interference control, and response inhibition.**

**However, this discussion has now been expanded to explicitly acknowledge the lack of formal validation of the modified tasks against other more traditional measures as a limitation, and the need for future work to further establish their construct validity (p. 34).**

e) Outcome Metrics: While reaction time (RT) and accuracy are standard metrics, the study does not address potential trade-offs between these measures (e.g., participants sacrificing speed for accuracy). This omission could distort interpretations of EF performance.

**Thank you for this helpful observation. We agree that potential speed–accuracy trade-offs are important to consider when interpreting executive function performance. Although the pattern of results in the accuracy analyses did not contradict those in the RT analyses, we have conducted additional analyses to assess possible trade-off effects. These are now presented in Supplementary Materials S2 and S5. In brief, the results showed no evidence of a speed–accuracy trade-off in any task or condition.**

- **Statistical Analysis**

a) High Variability in Results: The reported standard deviations (e.g., SD = 140ms in high-memory/incongruent conditions) are unusually high relative to the means, suggesting substantial uncontrolled variability that weakens the robustness of inferential statistics.

**Thank you for this thoughtful comment. We would like to clarify that the RT data were already cleaned at the trial level using a robust and pre-registered trimming method. Specifically, we applied Median Absolute Deviation (MAD) trimming separately for each participant, within each task, memory condition, and trial type (congruent/incongruent).**

**However, in direct response to this comment, we further conducted a Mahalanobis distance analysis separately for each task within each study, based on the four RT trial types (2 memory × 2 inhibition). Participants whose values exceeded the critical threshold for p = 0.001 (χ² > 18.47, df = 4) were excluded. All related analyses have been updated accordingly after removing these cases within each task and each study.**

**Furthermore, we would like to note that standard deviations in the range of 130–140 ms are typical in cognitive tasks involving interference, particularly in incongruent conditions. For example, Medrano (2024), in ‘Flanker Task Performance in Young and Older Adults: A Behavioral and ERP Study’, and Duthoo & Notebaert (2012), in ‘Conflict Adaptation: It Is Not What You Expect’, report RT variability of similar magnitude. These findings reinforce our view the observed standard deviations in our study likely reflect genuine cognitive processing demands rather than uncontrolled noise.**

**Finally, we would like to emphasise that, even if the reported standard deviations appear relatively large, this can only limit our power to detect effects. Nevertheless, the main effects of interest were consistently observed across both studies, suggesting that the tasks remained sufficiently sensitive to capture the intended constructs. The variability in Study 2 likely reflects the more diverse and representative sample recruited through Prolific, as compared to the more homogeneous university sample in Study 1. This increased variability is not necessarily indicative of noise or error, but rather a reflection of natural variation in a broader population. This point has now been clarified in the revised discussion (p. 32).**

b) Lack of Control Variables: The study fails to include potential confounding variables such as IQ, baseline working memory capacity, or sensory sensitivities as covariates. These omissions make it difficult to attribute observed effects solely to the experimental manipulations.

**We acknowledge the value of including potential confounding variables. Indeed, in our design, the low-memory and congruent condition served as a within-subject baseline across all participants to compare performance in the other conditions. Furthermore, by comparing performance relative to this baseline, we are (although indirectly) controlling for stable individual differences, including general cognitive ability such as IQ. Since each participant acts as their own control, this approach reduces the influence of between-subject variability on the observed effects (a point we now make on p. 39).**

**However, given that we did not directly measure sensory sensitivities or other potentially confounding variables such as attention or emotional regulation, we have readily acknowledged this as a limitation in both the Discussion (p. 40) and Conclusion (p. 41) sections of the revised manuscript.**

c) Ambiguity in Bayesian Analysis: Although Bayesian ANOVA was employed, the manuscript does not address whether assumptions of data homogeneity and independence were satisfied. The lack of sensitivity analyses further undermines confidence in the reported Bayes factors.

**While the assumptions for Bayesian repeated measures ANOVA are somewhat less strict than those required for classical frequentist ANOVA, we confirm that the relevant assumptions, particularly the independence of observations, were met and have now been clearly stated in the revised manuscript (p. 17 and 26). Additionally, for repeated measures Bayesian ANOVA, the assumption of homogeneity of variance is replaced by the assumption of sphericity. In our design, with two within-subject’s factors each having only two levels, the assumption of sphericity is automatically satisfied and therefore not a concern.**

- **Interpretation of Results**

a) Non-Significant Interaction Effects: The absence of "over-additive" interactions between working memory and inhibitory control should have prompted a deeper exploration of task sensitivity limitations rather than being presented as an expected outcome. Additionally, alternative explanations, such as cognitive compensatory mechanisms, are not addressed.

**We acknowledge the potential for further improvements in task design,** **which has now been further clarified in the Discussion. However, particularly in Study 2, we observed robust main effects of our manipulations (albeit with the congruency effect being evident only under low memory load) indicating that the tasks were sensitive and the manipulations worked. The absence of an over-additive interaction therefore does not imply a lack of task sensitivity; rather, it suggests that working memory and response inhibition may operate in an under-additive or independent fashion under the current task demands.**

**Second, the use of Bayesian analysis in this study allows for a more rigorous interpretation of the absence of interaction than would be possible with traditional frequentist approaches. Rather than simply failing to detect an effect, the Bayes factors provide evidence against the presence/supporting the absence of an (over-additive) interaction.**

**Finally, this study was conducted in a general population sample and investigated ASC/ADHD traits dimensionally, in line with a transdiagnostic framework. While compensatory mechanisms may be relevant in certain clinical contexts, they are not necessarily expected in a typically developing population. Moreover, such mechanisms would likely reduce the magnitude of main effects, not specifically suppress interaction effects. Nevertheless, we observed clear and consistent main effects across tasks, further supporting the validity and sensitivity of our design.**

**That said, we acknowledge that we may have misunderstood the reviewer’s intention regarding compensatory mechanisms and apologise if this is the case. We are happy to clarify or expand on this point further if needed. Please let us know if any additional revisions would be helpful.**

b) Contradiction of the Transdiagnostic Model: Despite endorsing a transdiagnostic approach, the study relies heavily on categorical distinctions (e.g., ASC traits versus ADHD traits), undermining its theoretical coherence.

**The transdiagnostic approach we adopted is now explained more consistently throughout the revised manuscript, and the alignment of our analytic strategy with this framework has been clarified. While ASC and ADHD traits were examined separately, this was done using continuous measures within a general population sample, which aligns with the dimensional nature of transdiagnostic models.**

c) Limited Relevance to Neurodivergent Populations: As the findings are based exclusively on neurotypical participants, they do not directly inform our understanding of EF in neurodivergent populations. This translational gap is not adequately addressed in the discussion.

**Thank you for highlighting this important point. This paper adopted a transdiagnostic, dimensional approach by examining ASD and ADHD traits within the general population, rather than a traditional categorical approach. However, we acknowledge that the sample consisted of participants who were not recruited on the basis of any formal diagnosis, and we accept that this limits the direct applicability of the findings to neurodivergent populations. This limitation has now been addressed more explicitly in the Discussion section (p. 39).**

**Feedback from Reviewer 2**

Reviewer 2’s comments have been grouped into two main areas: feedback related to Study 1 and feedback related to Study 2. Each point is presented and addressed below, respectively.

- **Study 1**

a) In Study 1, In lines 212–216, the instructions appear contradictory (or at least unclear) when compared to lines 261–262. It is advisable to clarify this section to prevent potential confusion for readers.

**Thank you for pointing that out. This has now been addressed in the revised manuscript, and the correct and consistent rules have been clarified in the updated version (p. 13).**

b) Regarding the instrument used to measure autistic traits (AQS), in lines 273–275, the authors describe the domains assessed by this tool and a factor derived from the mentioned dimensions. However, they later refer to the total score of the instrument without prior explanation or clarification of what high or low scores signify. To improve reader comprehension, it is recommended to clarify how the total score is derived and what it represents.

Similarly, in line 349, where the mean total score of the AQS is reported, the meaning of this value remains unclear. The methods section should include a clear explanation of how the total score is calculated and its interpretation.

**Thank you for the helpful comment. These points related to the AQ-S have now been addressed in the revised manuscript (p. 16) by clarifying how the total score is derived and what it represents. The same clarifications have also been applied to the descriptions of the CATI and ASRS measures (p. 25).**

c) The abbreviation "BF" is not defined anywhere in the methods section. It is recommended to specify what this abbreviation stands for when it first appears.

**Thank you for pointing this out. "BF" (Bayes Factor) is now defined at its first mention in the Results section (of the study 1, p. 16) of the revised manuscript.**

- **Study 2**

a) The OSF platform material associated with Study 2, which was inaccessible to me.

**We apologise for that, and the updated and accessible links of both studies have now been included in the revised manuscript (p. 11 and 22).**

d) In Study 2, in the introduction section, line 388, the authors mention that the memory load conditions were increased from four to six items. However, it is unclear at this point what this entails or how the task with six items works. Since this aspect is described in the measures section, it would be helpful to clarify this earlier in the text and reference the section where the information is located.

**The increase from four to six memory items builds on the design from Study 1, where the memory manipulation and types of stimuli were previously explained. However, a brief clarification has now been added to the introduction of Study 2 (p. 22) for clarity, and the details were already described in the Measures section of this study.**

**General Notes**

- All references and the reference list have been thoroughly verified for accuracy. Zotero (version 7.0.15) was used to manage and format citations, ensuring consistency.
- Changes have been highlighted throughout. However, due to extensive revisions in the introduction, highlights were omitted, though Track Changes remains available.
