# Supplementary material for: Concurrent measurement of working memory and inhibitory control and their correlations with autistic and ADHD traits in the general population
Source: PLoS One. 2026 Jan 5;21(1):e0339846. doi: 10.1371/journal.pone.0339846 (PMC12768290; doi:10.1371/journal.pone.0339846)
Supplement: Attachment — Submitted filename: Response_to_Reviewers_auresp_2.docx [file pone.0339846.s017.docx]

**Introduction**

1. Although the authors have attempted to incorporate additional literature in response to reviewers, the flow of the introduction is jumpy and difficult to follow – it jumps back and forth between discussing executive function in general to how executive function is impaired in psychiatric populations. It may make sense to streamline the introduction – introduce the concept of executive function and its importance, particularly for psychiatric populations, then go into why it is difficult to measure (both from the psychiatric perspective and from the measurement perspective) and finally address how the current study fills those gaps.

Answer: The introduction has now been revised throughout to follow the recommended structure.

2. Similarly, the authors could be more clear in their definition of working memory and inhibitory control earlier in the introduction, as these are their primary operationalizations of executive function (they are referenced in lines 58-59 before they are defined later in the paragraph around lines 63-66).

Answer: This has now been moved earlier in the introduction, on page 3 (first page of the introduction), lines 44–51, to provide clearer and earlier definitions of working memory and inhibitory control.

3. I also noted throughout the introduction and the discussion section that the authors reference a variety of models for the structure of working memory and inhibitory control (the Norman and Shallice model and the Baddeley model in the introduction, and the Roberts and Pennington model in the discussion) – it would be helpful to have these all presented in the same section, as this is a question the present work attempts to adjudicate.

Answer: These models are now grouped together in the introduction (page 9, lines 164–197) and are briefly revisited in the relevant part of the discussion (page 42-43, lines 876–887).

4. the discussion of compensatory mechanisms may be more appropriate for the discussion section, especially when trying to explain the lack of a correlation between performance and psychiatric traits.

Answer: This discussion of compensatory mechanisms has now been moved to the discussion section (page 47, lines 984-993) to better explain the lack of correlation between performance and psychiatric traits.

5. Although the authors do provide some description of their hypotheses, the term “over-additive” is not one that I’ve come across and would benefit from some additional clarification.

Answer: Additional explanation of the term “over-additive” has been included on page 8 (lines 174–182).

6. It would be incredibly helpful if the authors included a more tangible hypothesis in terms of what changes one would expect in the differences in reaction time or accuracy across memory/inhibition conditions.

Answer: This has been addressed on page 10-11, lines 227–247, where we clearly outline the preregistered hypotheses, including the expected differences in reaction time, accuracy, and inverse efficiency scores across the memory and inhibition conditions.

**Some additional places where I did not understand what the authors were trying to say:**

1. Line 62 – I’m not quite clear on what a “supportive mechanism” is.

Answer: The sentence containing this term was removed as part of a larger edit to the introduction.

2. Line 88 – I did not know what the “general level of large, broad studies” – does this mean larger populations of individuals with ASD? Studies that include typically developing individuals? Wider age ranges?

Answer: This sentence was also deleted during the revision of the introduction.

3. Line 160 – it may be more clear to specify what kinds of sub-clinical traits can be present in non-formally diagnosed individuals, rather than simply stating “autistic traits,” especially given that autism is such a heterogenous disorder.

Answer: This has been clarified on page 6, lines 117–121.

4. Line 176 – individual differences and phenotypic overlap does not necessarily impact EF research as a whole (as written), but studying EF in psychiatric populations

Answer: This has been clarified on page 7, lines 139–140.

**Method**

**Task design**

1. It’s important to note that in Study 1, the high memory load condition could also be simplified to 2 rules – warm colours (yellow/red) vs cool colours (blue/purple).

Answer: We have now addressed this point in the Study 1 Methods section (p. 16, lines 361-364) by adding the following clarification:

*“It is important to note that this colour set could potentially be categorized as warm versus cool colours, which may have reduced the intended working memory demand. This limitation was addressed in Study 2, considering the complementary nature of the two studies; see Study 2.”*

2. What was the inter-trial interval? Did the task move between trials before a response was made? This is relevant because some of the responses were over a second long – if the response was longer than the ITI, it would make sense to trim/exclude.

Answer: There was no fixed inter-trial interval, as the task was fully response-initiated. The next trial began only after a response was made, and the task never advanced without participant input. Although participants were instructed to respond as quickly and accurately as possible, no external time limit was imposed. (Page 15, lines 346).

3. It would be helpful to briefly reiterate how response inhibition was manipulated in the spatial conflict task in Study 2 (rather than just saying it was the same as Study 1).

Answer: This has now been added and clearly explained (p. 29, line 594-599).

**Inclusion/exclusion criteria**

1. Were participants screened for color-blindness?

Answer: Yes. Before taking part in either study, all participants were asked to report whether they were colour-blind using the following screening question:

“Are you colour-blind? (Yes/No)”

This question was included with the intention of excluding colour-blind individuals, although no participants in either study reported being colour-blind. This information has now been added to the exclusion criteria sections of both studies (for Study 1: p.10, lines 441-443 and for Study 2: p.32, lines 667-679).

**Statistical Analyses**

1. The authors seem to reference to testing different models in the results section but do not provide any reference as to what models were tested (what were the full and restricted models?) and how they were compared. This should be explicitly outlined.

Answer: In the Bayesian repeated-measures ANOVA, we compared a set of models: a full model (with both main effects and their interaction), restricted models (lacking one or more effects), and a null model (including only subject effects and random slopes). We computed Bayes Factors to compare all models against the null model (BF₁₀). Furthermore, we derived inclusion Bayes Factors from this model set to quantify the evidence for each effect. The same model comparison approach was used for the Bayesian correlations and regressions. More detailed information is now provided in each relevant results sections.

2. Second, the data, as presented in tables and line charts, obscures the underlying distribution of data and the individual variability of the performance and the psychiatric traits. It would be helpful to have the accuracy/RT data presented as bar charts (rather than line plots), with dots reflecting mean performance/RT for each individual participant.

Answer: These figures (as well as the one for IE) have now been updated to violin plots with individual participant dots to better show the underlying distribution and variability.

3. Moreover, the correlation plots of reaction time vs ASD/ADHD trait in Appendix S3 and S7 should be included as main figures – they allow the reader to inspect the distribution of performance and psychiatric traits.

Answer: We appreciate the suggestion. However, as the analyses did not provide evidence for the proposed associations, we believe these plots are more appropriately kept in the Supplementary Materials rather than moved to the main text.

4. It would also be helpful for the interpretation of the relationship (or lack thereof) between psychiatric trait and executive function to note whether there is a threshold that implicates diagnosis (i.e. if the AQ-S has a range of scores from 28 to 112, would we expect someone who is diagnosed with ASD to score 50? 100?). Having this information helps contextualize the results and how they might generalize past the general population. It may also make the plots more intuitive to interpret if the trait score is on the x-axis and the performance measure is on the y-axis.

Answer: Although this study focuses on autistic and ADHD traits within the general population and adopts a transdiagnostic rather than categorical diagnostic approach, we now include the relevant cut-off information and mark these thresholds in the correlational graphs. We have also placed trait scores on the x-axis and performance measures on the y-axis (see S3 Appendix for Study 1, S9 Appendix for ASC in Study 2, and S10 Appendix for ADHD in Study 2).

5. For the correlation analyses, the posterior mean correlation and 95% credible interval for the correlations should be provided, in addition to the Bayes Factor.

Answer: We now report the posterior mean correlations and their 95% credible intervals alongside the Bayes Factors for each zero-order correlation (see S3 Appendix for Study 1, S9 Appendix for ASC in Study 2, and S10 Appendix for ADHD in Study 2).

6. More detail should also be provided about the how the partial correlation analyses were conducted – were these true partial correlations, where the control variable (low working memory load or congruent trial condition) was regressed out of the outcome and then correlations were performed, or was a Bayesian linear regression performed (as was implied in a section heading from the Results section)? Either way, information about the posterior mean distribution and 95% credible interval should be provided in addition to the Bayes Factor (as mentioned for the zero-order correlations).

Answer: They were conducted as Bayesian regression analyses (equivalent to partial correlations), and this is now stated clearly. We also report the posterior mean estimates and their 95% credible intervals alongside the Bayes Factors for these analyses (see S4 Appendix for Study 1 and S11 Appendix for ASC and S13 Appendix for ADHD in Study 2).

7. Finally, although the authors have added in an additional supplementary analyses regarding a speed accuracy trade-off, the analyses they completed do not quite hit the mark – one could presumably respond slower to ensure accurate performance on even the low memory load/congruent conditions. It would be more appropriate to calculate inverse efficiency (IE = RT/proportion correct responses) and **use that as a dependent variable for analyses.** This may be more sensitive to relationships with psychiatric trait, especially given potential for compensatory mechanisms in psychiatric populations.

Answer: We have now calculated inverse efficiency (IE = RT / proportion correct) and included it as an additional dependent variable in all relevant analyses. IE results (to assess potential accuracy–RT trade-offs) are provided in S2 Appendix for Study 1 and S7 Appendix for Study 2. Furthermore, IE is now used alongside RT and accuracy in all related correlation analyses, including both zero-order and partial correlations for each task in each study. The IE results indicated no speed–accuracy trade-off, and overall, IE almost mirrored the RT pattern.

8. One additional point – the authors mentioned that there were various **subscales** for the AQ-S and the ASRS – was the relationships between these sub-scores and the EF measures investigated at all? I could imagine that EF may be more relevant for certain diagnostic criteria for autism, or that the core diagnostic questions for the ASRS may be more relevant.

Answer: Subscale analyses are now reported in S3 Appendix for Study 1 and in S11 Appendix (CATI) and S13 Appendix (ASRS) for Study 2. These analyses revealed no evidence of meaningful correlations between the size of any congruency or memory effects and higher scores on any ASC or ADHD questionnaire subscales.

9. Particularly for the partial correlation analyses, were **age and gender** included as covariates of no interest? I could imagine that age may be particularly relevant, especially given literature that age is negatively correlated with working memory capacity, and developmental changes in EF in children.

Answer: Age and gender were not included as covariates in the preregistration, as all participants were adults within a narrow age range (18–25 years), minimising variability related to developmental or age-related differences in executive function. However, in response to the reviewer’s request, we conducted an additional set of non-preregistered analyses including age and gender as covariates (see S12 Appendix for ASC and S14 Appendix for ADHD). These Bayesian regressions showed the same null pattern as the original analyses.

**Discussion**

1. After a cursory description of the inhibitory control constructs that the two tasks measure (Flanker task – interference control; spatial conflict – response inhibition), the differences are not discussed again and result are lumped into broader statements about inhibitory control. Especially given somewhat conflicting results across studies and tasks, and the framing in the introduction of inhibitory control being a multi-faceted construct, it may be worthwhile to discuss the differences in what the tasks are measuring and how that may have influenced results.

Answer: This has been addressed throughout the discussion, where we now directly mention the specific type of inhibition being referred to in each relevant section. Furthermore, specifically, on page 41, lines 833-848, we note that the differences in the main effects observed between the two tasks may be related to the different types of inhibition they involve.

2. The discussion of Hedge’s paradox (lines 839-841) may be more appropriate placed around discussion of the sensitivity of the task.

Answer: We appreciate the suggestion and have given it careful thought, but we believe this point fits best in its current position in the discussion, where it directly supports the interpretation of the lack of correlation between the cognitive task and questionnaire-based measures of executive function.

3. The authors do a nice job explaining why the congruency effect only appeared in the low memory condition in the Flanker task in Study 2; it would be helpful to also have some speculation as to why there memory effect in the spatial conflict task in Study 1 was only present in incongruent trials (as this seems less intuitive as to why it might have occurred).

Answer: This memory effect occurred in the reverse direction (participants were faster in the high-load than the low-load condition, as now noted on p. 22, lines 481-483). One possible explanation for this counter-intuitive pattern relates to the memory-load manipulation. In the low-load condition, the four stimuli were designed to be grouped into two pairs, whereas in the high-load condition they were intended to be non-groupable. However, as the reviewer highlighted, in the high-load condition W was mapped to blue and purple and P to red and yellow, which may have encouraged categorical grouping into *cool* versus *warm* colours. This unintended categorisation could have reduced the expected working-memory demands of the high-load condition (now clarified in the manuscript, p. 40, line 823-829).

**Throughout**

1. One thing that may help is being more structured with the various terminology – for instance, in the first sentence of the manuscript, the authors equate executive function to cognitive control. Later in in introduction in the discussion of the Norman and Shallice (1986) model, executive function and cognitive control seem to be described as two different constructs.

Answer: Thank you for pointing that out. The term ‘executive function’ is now used consistently throughout the manuscript wherever that meaning was intended, ensuring clarity and alignment in terminology.

**A few final points of clarification:**

1. What priors were used for all Bayesian analyses?

Answer: All analyses were conducted in JASP version 0.17.3 [95] using the default Cauchy priors on standardised effect sizes (r = 0.5 for fixed effects, r = 1 for random effects, and r = 0.354 for covariates). This is now added in the analysis plan for both studies (page 18 for Study 1 and page 31 for Study 2).

2. Were RT data log transformed prior to analysis (especially relevant given RT data are positively skewed) – this may also impact which priors should be used.

Answer: RT and IE data were log-transformed for the revised analyses. We used the default priors, as recommended by the JASP documentation:
*“When using Bayesian Repeated Measures ANOVA for log-transformed data, it is recommended to use the default prior, which allows for the possibility of individual differences in the magnitude of effects.”*

3. How many trials per participant were trimmed due to being classified as an outlier RT? Do results replicate if outlier trials are removed, versus being trimmed?

Answer: There was a confusion on our part in the earlier wording. Based on our preregistration plan, outlier RT trials were removed from all relevant analyses, not trimmed. Because trimming was not preregistered, we did not conduct additional trimming-based analyses.

This clarification is now clearly explained in the *Outliers and Exclusions* section of both studies.

4. For Table S7a, what does the column heading “Valid” mean?

Answer: It referred to the number of participants included in each analysis. This format has now been revised, and the exact number of participants is provided directly in each relevant descriptive statistics table.

5. It should be mentioned that random slopes (and potentially subject specific random intercepts) were included in the JASP models by default (if this option was not turned off during analysis).

Answer: Thank you for noting this. Random slopes and subject-specific random intercepts were included by default in the JASP models, as this option was not turned off. This is now stated in the analysis plan for both studies (page 17 for Study 1 and page 30 for Study 2).
